# *Arabidopsis* DEAD-Box RNA Helicase UAP56 Interacts with Both RNA and DNA as well as with mRNA Export Factors

**DOI:** 10.1371/journal.pone.0060644

**Published:** 2013-03-26

**Authors:** Christine Kammel, Maren Thomaier, Brian B. Sørensen, Thomas Schubert, Gernot Längst, Marion Grasser, Klaus D. Grasser

**Affiliations:** 1 Department of Cell Biology and Plant Biochemistry, Biochemie-Zentrum Regensburg, University of Regensburg, Regensburg, Germany; 2 Institute for Biochemistry III, Biochemie-Zentrum Regensburg, University of Regensburg, Regensburg, Germany; University of Toronto, Canada

## Abstract

The DEAD-box protein UAP56 (U2AF65-associcated protein) is an RNA helicase that in yeast and metazoa is critically involved in mRNA splicing and export. In *Arabidopsis*, two adjacent genes code for an identical UAP56 protein, and both genes are expressed. In case one of the genes is inactivated by a T-DNA insertion, wild type transcript level is maintained by the other intact gene. In contrast to other organisms that are severely affected by elevated UAP56 levels, *Arabidopsis* plants that overexpress UAP56 have wild type appearance. UAP56 localises predominantly to euchromatic regions of *Arabidopsis* nuclei, and associates with genes transcribed by RNA polymerase II independently from the presence of introns, while it is not detected at non-transcribed loci. Biochemical characterisation revealed that in addition to ssRNA and dsRNA, UAP56 interacts with dsDNA, but not with ssDNA. Moreover, the enzyme displays ATPase activity that is stimulated by RNA and dsDNA and it has ATP-dependent RNA helicase activity unwinding dsRNA, whereas it does not unwind dsDNA. Protein interaction studies showed that UAP56 directly interacts with the mRNA export factors ALY2 and MOS11, suggesting that it is involved in mRNA export from plant cell nuclei.

## Introduction

Nuclear pre-mRNA is extensively processed before it is exported to the cytosol for translation and splicing represents a central step in the mRNA maturation process. Assembly of the splicing machinery is mediated by a range of splicing factors including RNA helicases of the DEAD-box protein family, which belong to the superfamily 2 (SF2) of helicases [1]. Structural studies have shown that their conserved helicase core is often surrounded by variable auxiliary domains, which may be critical for the diverse functions of these enzymes. Typically, the helicase core has a series of conserved sequence motifs that play roles in ATP-binding, ATP-hydrolysis, RNA-binding, as well as dsRNA unwinding [2], [3]. DEAD-box helicases use ATP hydrolysis to mediate a variety of RNA structural rearrangements that are crucial during splicing and other RNA-dependent processes [2], [3].

In mammals, the 56-kDa DEAD-box RNA helicase UAP56 is an essential splicing factor that originally was identified as U2AF^65^-associated protein prompting its name UAP56. U2AF^65^ recruits UAP56 to the mRNA, where it is required for stable interaction between U2 snRNP and the pre-mRNA branch point [4]. Subsequently, it was found that UAP56 and its yeast counterpart termed SUB2 are involved in several steps of spliceosome assembly and their ATPase and helicase activities are required for that [5], [6]. Biochemical characterisation of human UAP56 revealed that it has RNA-stimulated ATPase activity and ATP-dependent RNA helicase activity [7]. Studies in different experimental systems have shown that UAP56 plays also an important role in the nuclear export of the mRNA [8]–[12]. Here UAP56 recruits the mRNA export adaptor ALY (Yra1 in yeast) to the spliced mRNA, coupling mRNA splicing and export. Moreover, human UAP56 can interact with another mRNA export protein termed CIP29 [13]. An additional interactor of UAP56 that contributes to mRNA export is the multiprotein complex THO. THO associates with UAP56 and ALY forming the TREX (transcription-export) complex and mutations in its subunits cause nuclear mRNA accumulation [14]. Accordingly, TREX contributes to the coordination of different steps in the mRNA biogenesis including transcription, processing and export [15], [16].

In plants, RNA helicase gene families are larger and more diversified than in other systems. The *Arabidopsis* genome, for instance, encodes a total of 113 helicases and 58 of them are putative members of the DEAD-box family of RNA helicases [17], [18], but only few of them have been experimentally analysed. Initially, genes encoding the DEAD-box family of eIF-4A translation initiation factors were identified that are differentially expressed in tobacco [19], [20]. Genes coding for various DEAD-box proteins were found to be required for proper development in *Arabidopsis* and *Brachypodium* [21]–[23], while others seem to play a role in plant responses to different types of abiotic stress and pathogen infection [24]–[28]. Recently, rice UAP56 (termed AIP1/2) was identified and it is involved in the degeneration of the tapetum during anther development [29]. RNA helicase activity was biochemically demonstrated for two plant DEAD-box proteins, rice OsBIRH1 and *Arabidopsis* AtDRH1 [28], [30], which are only distantly related (∼20% amino acid sequence identity) to UAP56. Here, we have analysed *Arabidopsis* UAP56, which is encoded by two genes. The *Arabidopsis* protein has RNA-stimulated ATPase activity and ATP-dependent RNA unwinding activity. Moreover, it binds both RNA and dsDNA, and it interacts with the mRNA export factors ALY2 and MOS11.

## Materials and Methods

### Plant material

*Arabidopsis thaliana* (Col-0) and the T-DNA insertion lines from the SALK collection [31] kindly provided by Nottingham Arabidopsis Stock Centre (NASC, http://arabidopsis.info/) were used. After sowing seeds were stratified (48 h at 4°C) and plants were grown in soil in a phytochamber at 22°C and 16 h photoperiods (LD) at ∼7,000 lux [32]. Transgenic plants were generated using *Agrobacterium*-mediated transformation as previously described [32], [33].

### PCR-based genotyping of plant lines and reverse-transcribed PCR (rtPCR)

The presence of T-DNA insertions was analysed by PCR-based genotyping [34]. Genomic DNA was isolated from leaves and was used for PCR analysis with *Taq* DNA polymerase (Peqlab) and primers specific for DNA insertions and the target genes (for primer sequences, see Table S1). For rtPCR, total RNA was extracted from ∼100 mg of frozen plant tissue using the TRIzol method (Invitrogen), before the RNA samples were treated with DNAse (MBI Fermentas). Reverse transcription was performed using 1 µg of RNA, random primers and Revert Aid H minus M-MuLV reverse transcriptase (MBI Fermentas). The obtained cDNA was used for PCR analyses using *Taq* DNA polymerase (Peqlab).

### Plasmid constructions

The coding sequence (CDS) of UAP56 was amplified by PCR with *Pfu* DNA polymerase using an *Arabidopsis thaliana* cDNA library as template and the primers (providing also the required restriction enzyme cleavage sites) listed in Table S1. The PCR fragments were inserted into suitable plasmids as detailed below. For transient expression of a GFP-UAP56 fusion in protoplasts, the coding sequence was inserted into plasmid p3′GFP [35] (providing the expressed protein with a C-terminal green fluorescent protein (GFP) fusion). For expression of UAP56 in *E. coli*, the CDS was inserted into plasmid pQE9 (Qiagen) providing an N-terminal 6×His-tag and into plasmid pGEX-5X-1 (Amersham) providing an N-terminal glutathione S transferase (GST) fusion. For overexpression in plants, the CDS was inserted under control of the CaMV 35 S promoter into pGreenII0229myc providing an N-terminal myc-tag. The CDS of ALY2 was inserted into pBluescript (Stratagene) and the CDS of MOS11 into pQE9. All plasmid constructions were checked by DNA sequencing, and details of the plasmids generated in this work are summarized in Table S1.

### Production of proteins

*E. coli* M15 cells were transformed with the pQE9-UAP56 and pQE9-MOS11 expression vectors and the recombinant 6xHis-tagged UAP56 and MOS11 were purified by metal-chelate column chromatography using Ni-NTA agarose (Qiagen) from *E. coli* lysates essentially as described previously [36] with minor modifications. After protein binding, the column was thoroughly washed using a buffer containing (50 mM Tris-HCl, pH 7.5, 1 M NaCl, 10% (v/v) glycerol, 10 mM 2-mercaptoethanol, 0.5 mM PMSF, 100 mg/ml benzamidine, 10 mM imidazole) and eluted with the same buffer containing 350 mM imidazole. The production of 6xHis-tagged HMGB2 was previously described [37]. GST and GST-UAP56 were expressed in *E. coli* using the pGEX-5X-1and pGEX-5X-1-UAP56 vectors, respectively, and purified by glutathione-sepharose affinity chromatography as previously described [38]. Using PD10 columns (Pharmacia) the buffer containing the purified proteins was changed to (10 mM phosphate buffer, pH 7.0, 1 mM EDTA, 1 mM DTT, 0.5 mM PMSF) and the recombinant proteins were analysed by SDS-PAGE and MALDI-TOF mass spectrometry. Since we were unable to produce 6xHis-tagged ALY2 in *E. coli*, it was synthesized as a ^35^S-methione-labelled protein using plasmid pBluescript-ALY2 and a coupled *in vitro* transcription/translation system (TNT coupled wheat germ extract system, Promega).

### Immunoblotting and immunofluorescence analyses

An antiserum against purified recombinant *Arabidopsis* 6xHis-UAP56 was produced by commercial immunisation and tested as previously described [32]. Protein extracts from leaves of three week-old plants were prepared as previously described [39] and analysed by immunoblot analyses [40] using the UAP56 antiserum. For immunofluorescence analyses sample preparation and antibody binding was performed as previously described [33], [40]. In addition to the UAP56 antiserum (diluted 1∶300), commercial monoclonal antibodies specific for the C-terminal domain of RNA polymerase II (Abcam, ab817, diluted 1∶100) and for fibrillarin (Abcam, ab4566, diluted 1∶300) were used as well as DAPI counterstaining. As secondary antibodies CY3-conjugated anti-rabbit IgG and fluorescein isothiocyanate (FITC)-conjugated anti-mouse IgG (Dianova) were used. Immunofluorescence images were recorded with an Axio Scope fluorescence microscope (Zeiss) equipped with an AxioCam MRm camera (Zeiss).

### Whole mount *in situ* mRNA localisation

To detect the localisation of total mRNA, we used an Alexa-488 end-labeled 48-mer oligo d(T) probe (MWG) as previously described [41].

### Transient protoplast transformation assays with GFP fusion constructs

Protoplasts of dark-grown tobacco BY-2 cells were transiently transformed using PEG-mediated transformation and analysed for the localisation of GFP fusion proteins by confocal laser scanning microscopy (CLSM) as previously described [32], [40] using a LSM 510 microscope (Zeiss).

### Microscale thermophoresis (MST) and electrophoretic mobility shift assays (EMSAs)

Protein interactions with DNA and RNA oligonucleotides were analysed by MST essentially as previously described [42], [43]. In brief, binding reactions containing 50 nM fluorescently labelled RNA or DNA (see Table S2) in EMSA buffer [44] and various concentrations of 6xHis-UAP56 were analysed in standard capillaries using the Monolith NT.015T MST-instrument (NanoTemper) at 28°C. The recorded fluorescence is normalised and processed using the software OriginPro 8.5 (OriginLab) and fitted using the Hill equation as previously described [43]. Under these experimental conditions, K_d_ corresponds to the protein concentration resulting in binding of 50% of the oligonucleotides. In addition, protein interactions with radio-labelled RNA and DNA oligonucleotides were examined by EMSAs as previously described [44]. In brief, different protein concentrations were incubated with the ^32^P-labelled RNA or DNA probe (see Table S2) and protein binding was analysed by polyacrylamide gel electrophoresis followed by phosphorimaging.

### Chromatin immunoprecipitation (ChIP)

ChIP experiments were performed essentially as previously described [34] using Col-0 plants (14 days after stratification, DAS). In addition to the UAP56 antibody and the corresponding preimmune serum, an antibody against the C-terminal region of H3 (ab1791, Abcam) and against SPT16 [45] were used. DNA was analysed by PCR with locus-specific primers (Table S1), and the amount of template was adjusted for amplification efficiency relative to the DNA purified from chromatin prior to immunoprecipitation (input). Thus, due to the different DNA concentrations of the different samples, for SPT16, UAP56 and mock (no antibody added) twice the amount of template was used relative to the H3 sample, and four-times the amount relative to the input DNA. ChIP experiments were repeated three-times.

### ATPase assay

ATPase assays were essentially performed as previously described [7]. The reaction was started by addition of 6xHis-UAP56 (or HMGB2 control protein) to the reaction mixture containing (50 mM Tris-HCl, pH 8.5, 50 mM KCl, 2 mM MgCl_2_, 0.1 mg/ml BSA, 1 mM DTT, 10 µM unlabelled ATP, 2 µCi [α-^32^P]ATP) and different amounts of synthetic RNA oligonucleotides (MWG). The reaction in a final volume of 20 µl was allowed to proceed at 25°C for 30 min and was stopped by adding EDTA to a final concentration of 20 mM. ATP was separated from ADP by thin layer chromatography (TLC). Therefore 5 µl of the reaction mix was spotted onto polyethyleneimin cellulose TLC plates (Merck), which was developed in a buffer containing (2 M acetic acid, 0.5 M LiCl_2_). The plate was dried and analysed by phosphorimaging.

### RNA helicase assay

RNA helicase/unwinding assays were essentially performed as previously described [7]. One strand of the RNA substrate was labelled at the 5′-end with [𝛘-^32^P]ATP (Hartmann Analytic) using T4 polynucleotide kinase (Fermentas). The reaction mix was boiled for 5 min to inactivate the kinase. The complementary unlabeled strand was added in a 1∶1.2 molar ratio (labelled to unlabelled). For annealing, the mixture was heated to 100°C for 5 min and then slowly cooled to 25°C. The labelled dsRNA was then purified by using G25 columns (GE Healthcare). The helicase assay was started by addition of 6xHis-UAP56 to the reaction mixture containing (50 mM Tris-HCl, pH 8.5, 50 mM KCl, 2 mM MgCl2, 0.1 mg/ml BSA, 1 mM DTT, 5 U RiboLock) and 3 mM ATP (unless stated otherwise) and 2.5 nM dsRNA. After incubation at 28 °C for 1 h, the reaction was stopped by adding 5 µl stop solution (0.5% SDS, 50% glycerol, 100 mM EDTA, 0.1% bromphenol blue). After Proteinase K treatment, the unwound ssRNA was separated from the dsRNA substrate by electrophoresis on 16% polyacrylamide gels in 1xTBE. The dried gels were analysed by phosphorimaging.

### Protein-protein interaction assays

Yeast two-hybrid assays were essentially performed according to the manufacturer (Clontech). In brief, yeast cells of the strain AH109 were cotransformed with pGBKT7 and pGADT7 plasmids and grown at 30 °C on SD/-Leu/-Trp. For interaction assays, cells were grown on SD/-Leu/-Trp/-His medium for 2 d. For β-galactosidase assays cells were grown on the same medium and enzyme activity was detected after incubating the filters of colony lifts in a solution containing X-gal. As positive and negative controls served the interactions between p53 and the SV40 large T-antigen, and between lamin and the SV40 large T-antigen, respectively, provided by the manufacturer. Pull-down assays with GST and GST-UAP56 were performed as previously described [38] adapting published buffer conditions [5], [13] for the interaction with MOS11, HMGB2 and ALY2.

## Results

### Two *Arabidopsis* genes code for UAP56

To identify a possible ortholog of human UAP56 and yeast SUB2, we searched the *Arabidopsis* database (http://www.arabidopsis.org/) with the BLASTP program using these two amino acid sequences as query. The search resulted in two prominent hits. Despite some variation of the adjacent loci (At5g11170, At5g11200) in the nucleotide sequences of the transcribed regions, the two closely related genes code for an identical UAP56 amino acid sequence of 427 residues (48,3 kDa). Aligning the *Arabidopsis* UAP56 sequence with the amino acid sequences of UAP56 proteins of other organisms revealed that the characteristic helicase motifs [2], [3], [46] are well conserved including motif I (which is involved in ATP-binding), motif II (which is involved in ATP hydrolysis) and motifs IV and V (which are involved in RNA binding) (Figure S1). *Arabidopsis* UAP56 shares 89%, 70% and 61% amino acid sequence identity with rice UAP56, human UAP56 and yeast SUB2, respectively. Several plant genomes, for instance, of the monocot plant *Oryza sativa* and the dicot plant *Populus trichocarpa* have two genes encoding almost identical UAP56 proteins with ∼99% amino acid sequence identity. The relationship of (putative) UAP56 sequences of various plants and of other organisms is illustrated by a sequence similarity tree (Figure S2).

**Figure 1:**
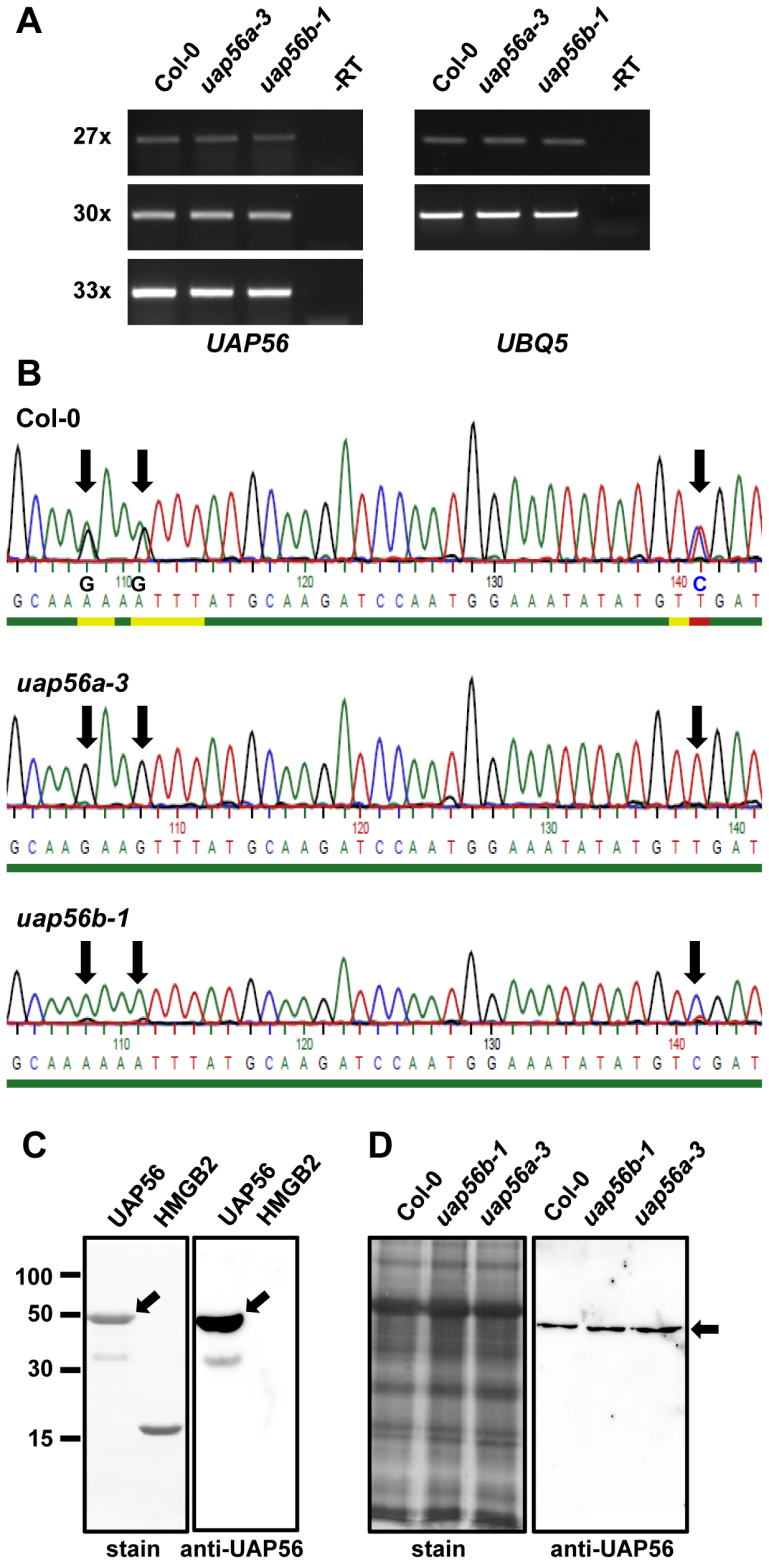
Expression of the *UAP56* genes. **A** rtPCR analysis of RNA isolated from Col-0, *uap56a-3* and *uap56b-1* plants using primers P7 and P8 (cf. Figure S3), annealing at regions that are common to both *UAP56* genes. As a reference the transcript of the house keeping gene *UBQ5* was amplified and as negative control a reaction without addition of reverse transcriptase is shown. PCR amplification was allowed to proceed for a different number of cycles (27x, 30x, 33x). **B** The UAP56 sequences of the cDNA fragments amplified from the three genotypes (cf. Part **A** of this figure) were determined by DNA sequencing. Within the shown region, the sequences of *UAP56a* and *UAP56b* are identical except for the three positions indicated by arrows. **C** Production of recombinant UAP56 and an UAP56 antiserum. Analysis of purified recombinant UAP56 (and the reference protein HMGB2) by SDS-PAGE in a 12% polyacrylamide gel, which was stained with Coomassie (left panel). Immunoblot analysis of recombinant UAP56 (and HMGB2 as a reference) using the UAP56-antiserum (right panel). Full-length recombinant UAP56 is indicated by an arrow. **D** Comparable amounts of protein extracts of Col-0 and the two T-DNA insertion mutant lines were analysed by SDS-PAGE and Coomassie staining (left panel) and by immunoblot analysis using the UAP56-antiserum (right panel). *Arabidopsis* UAP56 detected by the antibody is indicated by an arrow.

We have examined several *Arabidopsis* T-DNA insertion mutant lines, to study the expression of the two *UAP56* genes and possible effects of the inactivation of these genes. One T-DNA insertion line for each gene was analysed in more detail (Figure S3A). In both cases, the T-DNA is inserted within intron sequences. As determined by PCR-based genotyping, we were able to isolate plants homozygous for the T-DNA insertion in At5g11170, termed *uap56a-3*, and in At5g11200, termed *uap56b-1*(Figure S3B). Plants of both mutant lines homozygous for the T-DNA insertion have wild type appearance (Figure S4). We examined the transcript levels of *UAP56a* and *UAP56b* in the aerial parts of three week-old plants by rtPCR, but due to the remarkable sequence similarity of the transcribed regions of the two genes, it is difficult to discriminate them reliably by PCR. For the amplification we used primers that match both genes to test the expression level in *uap56a-*3 and *uap56b-1*relative to the wild type Col-0. In plants of the three genotypes similar transcript levels of *UAP56* were detected (Figure 1A). Moreover, only a single DNA fragment was amplified, although according to the *Arabidopsis* database, within the amplified region there are alternative splice sites predicted. Hence, at least in the tested tissue, there is no indication of alternative splicing within this region of the *UAP56* genes. To distinguish the transcripts originating from the two *UAP56* genes, the DNA fragments amplified from the three genotypes (Figure 1A) were sequenced. Since there are few sequence differences between *UAP56a* and *UAP56b*, the nucleotide sequence obtained from Col-0 represents a mixture of the two sequences (Figure 1B), which is evident from the three sequence positions indicated by arrows. In contrast, the sequences obtained from *uap56a-*3 and *uap56b-1* represent only a single sequence each, corresponding to the non-mutated gene in these lines. This experiment demonstrates that in Col-0 indeed both *UAP56* genes are transcribed, whereas in the mutant lines only the transcript of a single gene is detectable. Nevertheless, the total transcript level is similar in the three genotypes (Figure 1A). This indicates that in the mutant lines the intact *UAP56* gene can compensate for the lacking transcript of the mutated gene, resulting in wild type transcript levels.

### Production of recombinant UAP56 and detection of UAP56 in *Arabidopsis*

To biochemically analyse *Arabidopsis* UAP56, we expressed a 6xHis-tagged protein in *E. coli*. The protein was soluble and could be purified efficiently by metal-chelate affinity chromatography. Analysis of the purified recombinant UAP56 by SDS-PAGE revealed that it is essentially pure, except for a weak contaminating band, migrating below the expected ∼50-kDa band of UAP56 (Figure 1C). Examination of the two protein bands by MALDI-TOF mass spectrometry identified the ∼50-kDa band as UAP56, while the band below most likely is a degradation product of UAP56. The recombinant protein was used for immunisation to raise antibodies against UAP56. The obtained antiserum reacted with recombinant UAP56 (and the band corresponding to the degradation product), but not with the control protein HMGB2 (Figure 1C). Protein extracts of leaves of the T-DNA insertion mutants *uap56a-*3and *uap56b-1*as well as of Col-0 control plants were analysed by immunoblot analysis using the UAP56-antiserum. The antibody detected a single protein band of ∼50 kDa (Figure 1D). Moreover, in line with the rtPCR results (Figure 1A), comparable amounts of the UAP56 protein were detected in the three genotypes. Since elevated levels of UAP56/SUB2 cause severe defects in *S. cerevisiae* and *C. elegans* [11], [12], we generated plants overexpressing myc-tagged UAP56 (Figure S5). However, plants overproducing UAP56 displayed wild type appearance throughout development and according to whole mount *in situ* mRNA localisation experiments their bulk mRNA distribution appears to be not affected (Figure S5).

**Figure 2:**
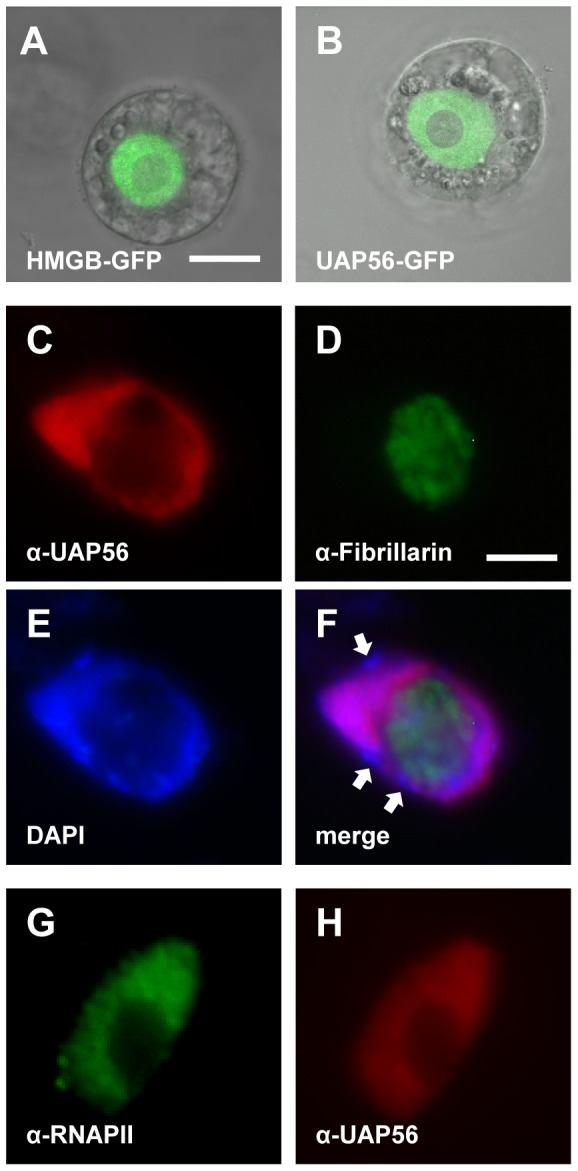
*Arabidopsis* UAP56 localises to the nucleus in protoplasts and root cells. Tobacco BY2 cell protoplasts were transformed with constructs driving the expression of the indicated GFP fusion proteins and GFP fluorescence was visualised by CLSM (**A,B**; size bar: 10 µm). GFP fluorescence of a nuclear HMGB protein and overlay with the corresponding bright field image (**A**). GFP fluorescence of a UAP56-GFP fusion (**B**). Analysis of *Arabidopsis* Col-0 root tip cells by immunofluorescence microscopy using different antibodies and DAPI staining (**E**–**H**, size bar: 5 µm). Immunostaining of fibrillarin and UAP56, as well as visualisation of the DNA by DAPI staining of the same nucleus (**C**–**E**). A merge of the three images is shown in (**F**) and examples of brightly DAPI-stained heterochromatic chromocenters are indicated by arrows. Immunostaining of RNAPII and UAP56 of the same nucleus (**G,H**).

We expressed an UAP56-GFP fusion protein in BY2 cell protoplasts, to examine the subcellular distribution of UAP56 by confocal laser scanning microscopy (CLSM). The chromosomal HMGB protein encoded by the At2g34450 locus that localises to the cell nucleus [44] served as a reference (Figure 2A). The UAP56-GFP fusion protein accumulated in the nucleus (Figure 2B), indicating that as in other organisms [8], [11], [47] *Arabidopsis* UAP56 is a nuclear protein. The nuclear localisation of native UAP56 was analysed by immunofluorescence microscopy of wild type *Arabidopsis* root tip cells. The UAP56 antiserum specifically reacted with nuclei (Figure 2C), confirming the results obtained with the GFP fusion proteins. The comparison with the nucleolar marker fibrillarin [27] in the same nucleus (Figure 2D) revealed that UAP56 localises predominantly to the nucleoplasm, but weak signals are also seen in the nucleolus. Comparison with the DNA dye, DAPI (Figure 2E), and the merge of the three individual stains (Figure 2F) indicates that UAP56 is more prominent in the less condensed euchromatin than in the condensed chromocenters. In line with that the nuclear distribution of RNA polymerase II (RNAPII) and UAP56 are similar (Figure 2G,H).

### UAP56 can interact with both RNA and dsDNA, and associates with transcribed loci

**Figure 3:**
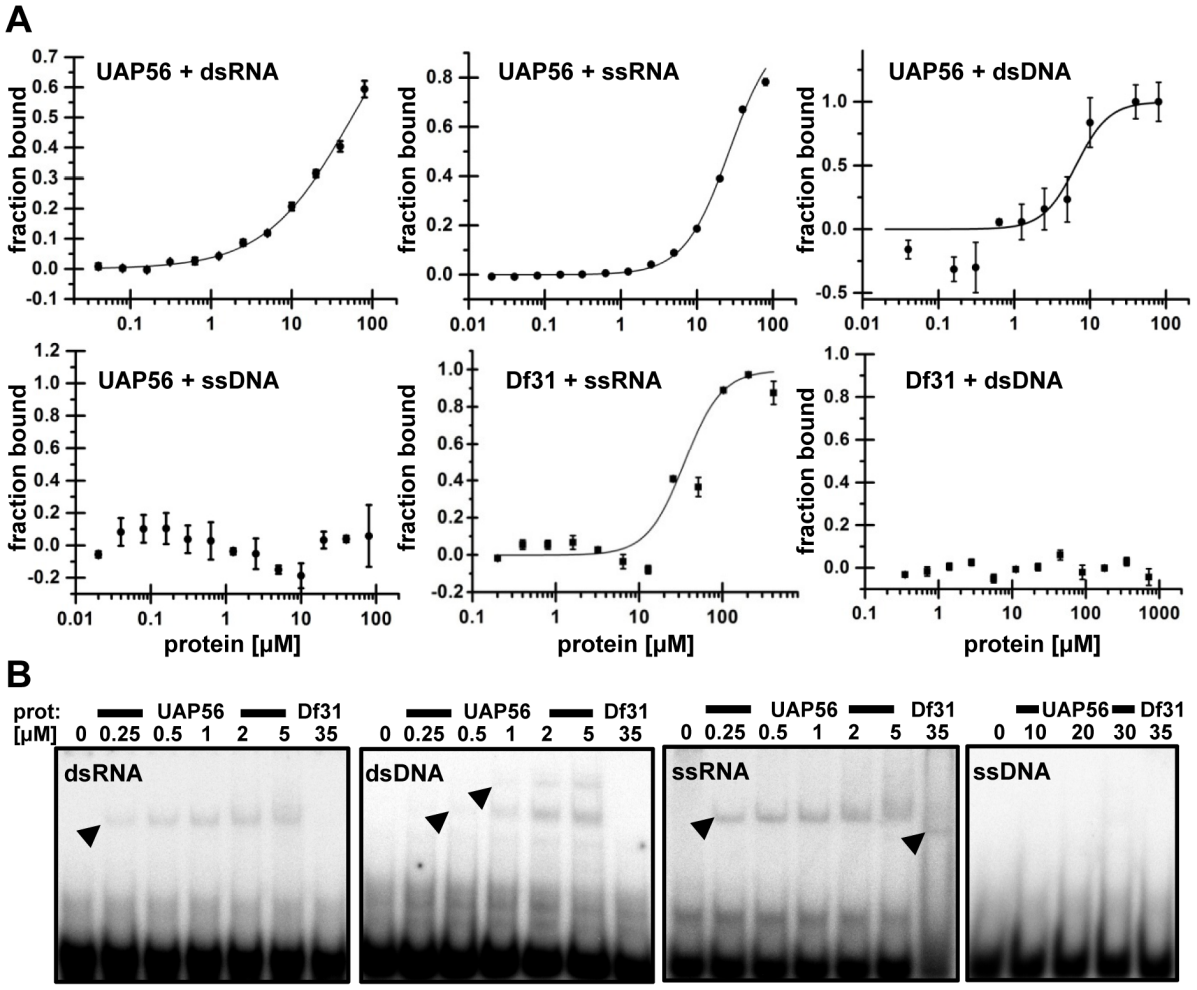
*Arabidopsis* UAP56 binds ssRNA, dsRNA and dsDNA, but not ssDNA. **A** Interaction with nucleic acids measured by MST. Fuorescently labelled 29-nt oligonucleotides of dsRNA, ssRNA, dsDNA and ssDNA were incubated with increasing concentrations (0.01–80 µM) of UAP56 (or as a reference with 0.2–411 µM Df31). Protein-nucleic acid interactions were quantified by MST and binding data are plotted using the Hill equation. Data represent the mean +/− SD of at least three technical replicates. **B** Interaction with nucleic acids analysed by EMSA. ^32^P-labelled 25-nt oligonucleotides were incubated without protein addition or with increasing concentrations (0.25–5 µM, except in case of ssDNA 10–30 µM) of UAP56 (or as a reference with 35 µM Df31). Protein binding was detected after electrophoresis by phosphorimaging and appearing protein-nucleic acid complexes are indicated by arrow heads.

To examine the interaction of recombinant *Arabidopsis* UAP56 with nucleic acids in solution, we used microscale thermophoresis (MST, [43]). The binding affinity was measured for fluorescently labelled 29-nt long oligonucleotides of ssRNA and dsRNA as well as for ssDNA and dsDNA. For comparison the *Drosophila* ssRNA-binding protein, Decondensation Factor 31 (Df31, [42]), was analysed in parallel (Figure 3A). UAP56 bound to both ssRNA and dsRNA with K_D_ values of 26.58 µM+/−3.11 and 51.45 µM +/−5.30, respectively. In view of the fact that SF2 helicases usually are strictly specific for RNA or DNA [1], it was surprising that UAP56 in addition to the RNA substrates clearly interacted with dsDNA (K_D_ = 6.5 µM+/−0.75), while ssDNA was not bound. As shown before by MST [42], Df31 interacted with ssRNA (Kd 35.29 µM+/−8.05) and failed to recognize dsDNA. The interaction of UAP56 (and Df31) with nucleic acids was additionally studied by electrophoretic mobility shift assays (EMSAs) using ^32^P-labelled 25-nt long oligonucleotides, which differed in sequence from the 29-nt oligonucleotides used for the MST analyses. As seen with MST, UAP56 bound to the ssRNA, dsRNA and dsDNA probes, and not to the ssDNA, while Df31 interacted exclusively with ssRNA (Figure 3B). With the dsDNA UAP56 formed two distinct complexes, while only a single complex was detected when the protein bound to RNA. Both MST and EMSA experiments show that UAP56 interacts with RNA and dsDNA, but there are quantitative differences between the results obtained with the two methods. Thus, higher protein concentrations are required to detect binding in the MST experiments, and the relative binding preferences differ to some extent. According to the MST measurements, UAP56 binds with higher affinity to dsDNA than to RNA, whereas the EMSAs indicate a more efficient binding to RNA. However, it should be stressed that EMSA and MST experiments were performed with oligonucleotides of different length and sequence. Moreover, in EMSAs the binding reaction occurs in solution, but it is well known that during gel electrophoresis complex stability/lifetime is influenced by various parameters most importantly the caging effect reducing the dissociation of protein-nucleic acid complexes [48]. By contrast MST measures interactions completely in solution. In conclusion, both the MST and the EMSA experiments showed that *Arabidopsis* UAP56 in addition to RNA can bind to dsDNA although DEAD-box helicases usually are considered RNA specific [2], [3].

**Figure 4:**
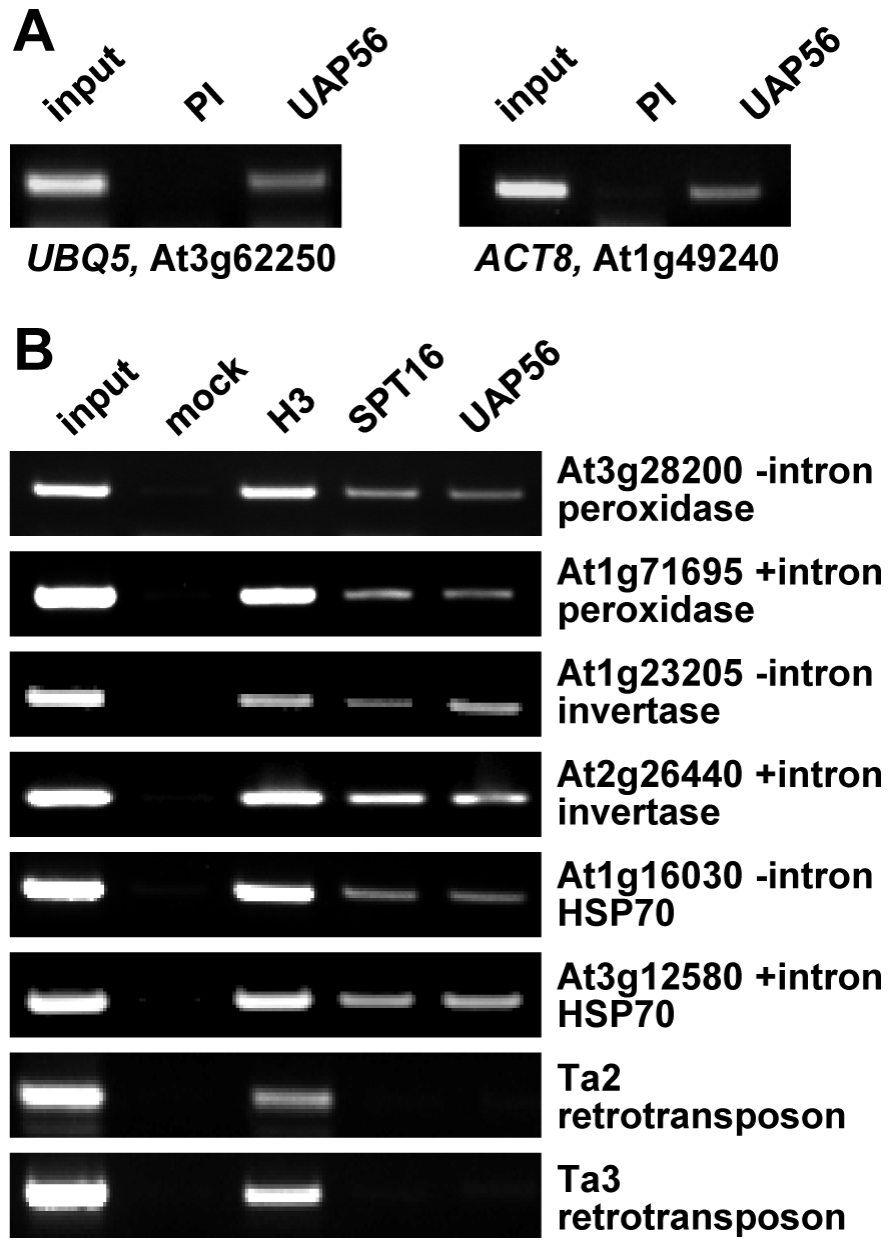
UAP56 associates with loci transcribed by RNA polymerase II independent from the presence of introns, but not with non-transcribed regions. A Chromatin was immunoprecipitated with the UAP56 antiserum and the corresponding preimmune serum (PI). DNA purified from the precipitated samples or input chromatin was examined by PCR using gene-specific primer combinations (cf. Table S1). B Chromatin was immunoprecipitated without addition of antibody (mock) or using antibodies against histone H3, the SPT16 subunit of FACT and UAP56. DNA purified from the precipitated samples was examined by PCR and the presence/absence of introns at the respective locus is indicated. For comparison two non-transcribed retrotransposons were analysed. Representative PCR analyses based on three independent ChIP experiments are shown.

We used chromatin immunoprecipitation (ChIP) to examine the association of UAP56 with RNAPII-transcribed loci in *Arabidopsis* plants. The UAP56 antiserum was compared with the preimmune serum in ChIP experiments analysing the association with the housekeeping genes *UBQ5* and *ACT8*. Gene-specific DNA fragments could be amplified by PCR using the input chromatin and the UAP56 precipitate as template, but not from the preimmune control sample (Figure 4A). Using the UAP56 antiserum and for comparison antibodies directed against histone H3 and the RNAPII transcript elongation factor SPT16 (a subunit of the FACT histone chaperone) selected genes were analysed by PCR following ChIP with the three antibodies. Each two genes (one intronless and one containing introns) encoding peroxidases, invertases and heat shock protein 70 (HSP70) were chosen for this analysis (Figure 4B). According to the *Arabidopsis* database (http://www.arabidopsis.org/) the selected genes are expressed in two week-old plants used for the ChIP experiment, which was confirmed by rtPCR (not shown). In addition to these transcribed genes, the association with the non-transcribed retrotransposons Ta2 and Ta3 [49] was examined. As expected, H3 was detected at all tested genomic regions. SPT16 was previously found to associate with transcribed loci, but not with non-transcribed regions [34], [45], and in line with that SPT16 was detected at the transcribed genes, but not at Ta2 and Ta3. Similar to SPT16, UAP56 associated with the transcribed regions independent from the presence/absence of introns, but not with the transcriptionally silent genomic loci (Figure 4B). The ChIP analyses indicate that in line with the immunfluorescence results (Figure 2), *Arabidopsis* UAP56 associates with sites of RNAPII transcription independent from mRNA splicing.

### ATPase and helicase activity of UAP56

**Figure 5:**
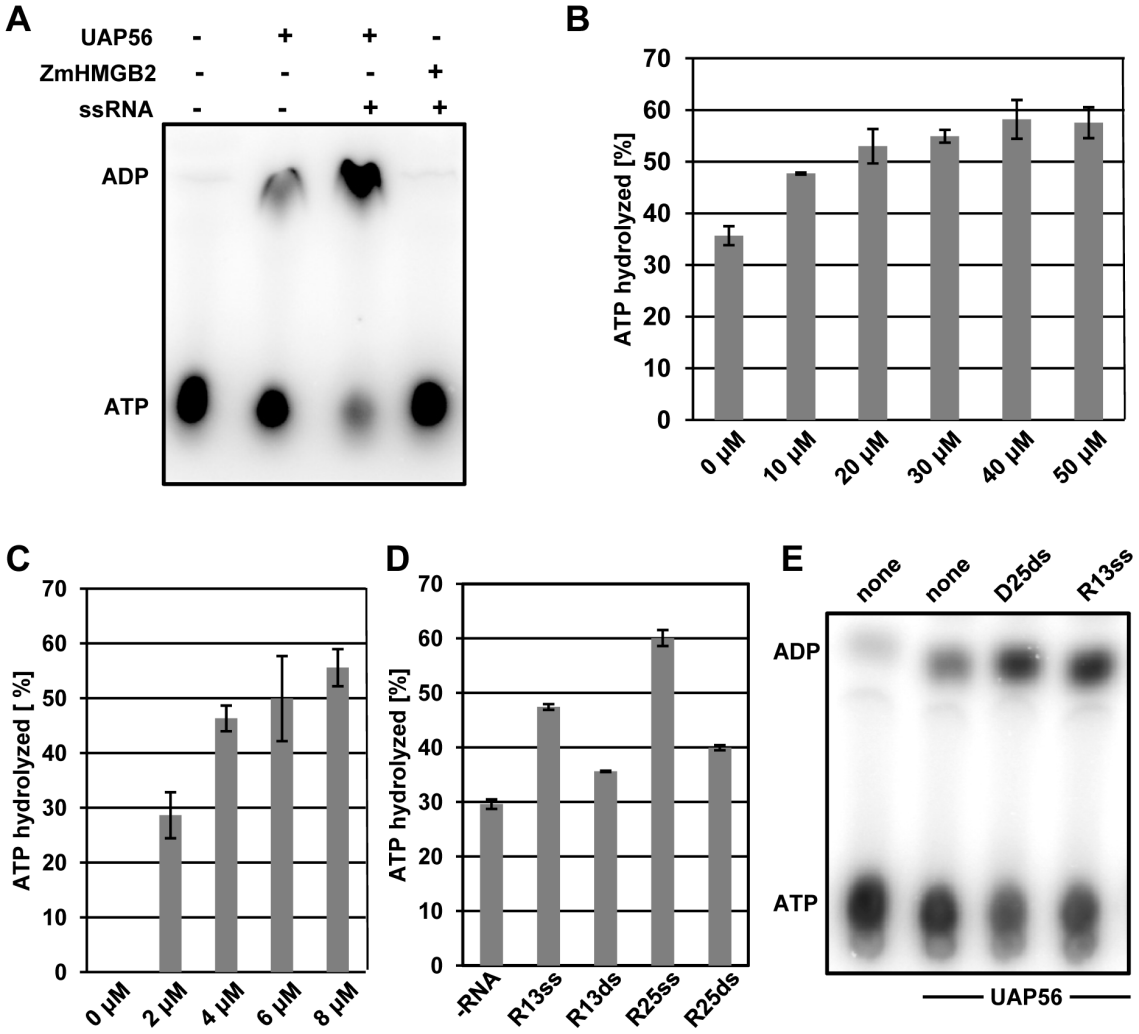
*Arabidopsis* UAP56 has ATPase acitivity that is stimulated by RNA and dsDNA. **A** ATP hydrolysis by UAP56 (or the control protein HMGB2, each 8 µM) was tested in the presence (absence) of ssRNA (R13, 50 µM). ATP and ADP were separated by TLC and analysed by phosphorimaging. **B** Stimulation of the ATPase activity of UAP56 (8 µM) by different amounts of ssRNA was quantified from three independently repeated experiments, and error bars indicate standard diviations. **C** ATPase activity of different concentrations of recombinant UAP56 in the presence of ssRNA (R13, 50 µM). **D** Stimulation of the ATPase activity by different RNAs (cf. Table S2). The effect of ssRNA vs. dsRNA and of short (13-nt) vs. longer (25-nt) RNA (50 µM each) on the ATPase activity of UAP56 (4 µM). As evaluated using Students t-test, the different RNAs stimulate the ATPase activity to different extents (P<0.05).**E** ATP hydrolysis of UAP56 in the absence of nucleic acids and in the presence of 13-nt ssRNA or 25-bp dsDNA.

UAP56 was examined *in vitro* for its catalytic activities. To study its ATP-hydrolysis activity, [α-^32^P]ATP (mixed with unlabelled ATP) was incubated with UAP56 or the chromosomal high mobility group HMGB2 protein, which served as a negative control. Afterwards ATP and ADP were separated by TLC and detected by phosphorimaging. In the absence of protein or in the presence of HMGB2 ATP hydrolysis was hardly detectable, whereas addition of UAP56 resulted in ATP hydrolysis, as evident from the clearly visible ADP spot (Figure 5A). When UAP56 and single-stranded RNA (ssRNA) was added to the reaction, ATP hydrolysis was stimulated, resulting in ∼60% increased amount of the ADP product and reduced amount of the ATP substrate. To further explore the ATPase activity of UAP56, ATP hydrolysis was analysed in the presence of increasing amounts of ssRNA (Figure 5B) and increasing amounts of UAP56 (Figure 5C). These experiments demonstrate that increasing ATP hydrolysis is observed in a dose-dependent manner with increasing input of UAP56 or ssRNA. We then examined ATP hydrolysis catalysed by UAP56 in the presence of different types of RNA (Table S2). 13-nt RNA was compared with 25-nt RNA and ssRNA was compared with dsRNA. Quantification of the ATP conversion in the presence of UAP56 and the different RNAs (Figure 5D) revealed that ssRNA is more efficient than dsRNA and the 25-nt RNA is more efficient than the 13-nt RNA in promoting ATP hydrolysis. In view of the interaction of UAP56 with dsDNA (Figure 3), it was tested whether dsDNA can stimulate the ATPase activity. Addition of 25-bp dsDNA (the same as used in Figure 3B) to the reaction resulted in stimulation of ATP hydrolysis that is similar to that seen with the 13-nt ssRNA (Figure 5E). Taken together these experiments show that *Arabidopsis* UAP56 has ATPase activity that is stimulated by RNA and dsDNA.

**Figure 6:**
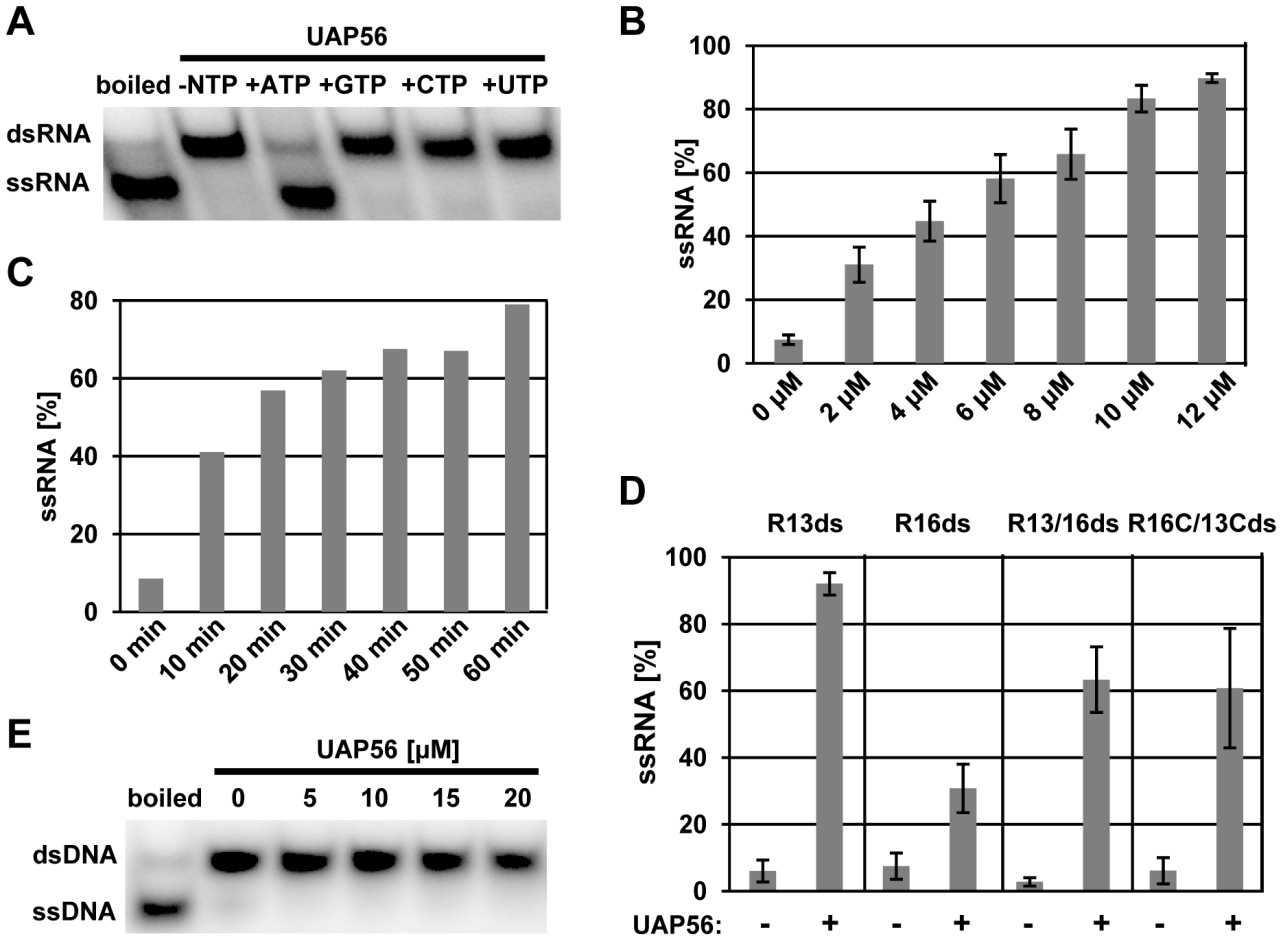
*Arabidopsis* UAP56 has ATP-dependent RNA helicase activity. **A** dsRNA unwinding activity of recombinant UAP56 (12 µM) in the presence of R13ds RNA in the absence or presence of different NTPs (3 mM, as indicated). dsRNA and ssRNA were separated by polyacrylamide electrophoresis and analysed by phosphorimaging. **B** The dsRNA unwinding activity of different amounts of recombinant UAP56 (0–12 µM) was analysed in the presence of ATP and a 13-nt dsRNA substrate (R13ds). Helicase activity was quantified from three independently repeated experiments, and error bars indicate the calculated standard diviations. **C** The dsRNA unwinding activity of 12 µM UAP56 was monitored for different times in the presence of ATP and a 13-nt dsRNA substrate (R13ds). **D** dsRNA unwinding activity of UAP56 (0 or 12 µM) in the presence of ATP and different dsRNAs (cf.Table S2). Helicase activity was quantified from three independently repeated experiments, and error bars indicate the calculated standard diviations. **E** The dsDNA unwinding activity of different amounts of recombinant UAP56 (0–20 µM) was analysed in the presence of ATP and a 13-nt dsDNA substrate (D13ds).

To examine the helicase activity of *Arabidopsis* UAP56, we tested whether the protein can unwind dsRNA. Boiling of the blunt-end 13-nt RNA duplex served as a positive control for the strand separation. The ^32^P-labelled dsRNA and ssRNA were separated by polyacrylamide gel electrophoresis and detected by phosphorimaging. We analysed the unwinding activity in the presence or absence of different nucleoside triphosphates. In the presence of ATP UAP56 clearly displayed unwinding activity, which is seen by the reduced amount of the dsRNA substrate and an increasing amount of the ssRNA product. In the absence of NTPs or in the presence of CTP, GTP or UTP no unwinding of the dsRNA could be detected (Figure 6A). This demonstrated that the unwinding activity of UAP56 is ATP-dependent. In the presence of ATP with increasing UAP56 protein input a protein concentration-dependent unwinding of the dsRNA is observed (Figure 6B). A time-course of the RNA unwinding assay revealed that the reaction progressed during 60 minutes (Figure 6C). The RNA helicase activity of UAP56 was further examined using various dsRNA substrates with different length and blunt-ends or single-strand overhangs (Table S2), since unwinding efficiency may depend on the length/stability of the substrate as well as the nature of its ends [2], [3]. Comparison of the unwinding of blunt-ended 13-nt and 16-nt RNAs (R13ds and R16ds, respectively) revealed that the shorter RNA substrate is unwound approximately threefold more efficiently than the longer version (Figure 6D). We also designed three RNA substrates with 13-nt base pairs that differed in their ends. The RNAs with 3′and 5′overhangs (R13/R16 and R13C/R16C, respectively) were unwound by UAP56 with comparable efficiency (∼60%), while the unwinding of the blunt-ended substrate (R13ds) was more efficient (∼90%, Figure 6C). Since dsDNA stimulated the ATPase activity of UAP56 (Figure 5E), we tested whether the protein displays also DNA helicase activity. When increasing amounts of UAP56 were incubated with a ^32^P-labelled 13-bp dsDNA probe (corresponding in sequence to R13ds used above) in the presence of ATP, no unwinding was observed (Figure 6E). Our experiments demonstrate that *Arabidopsis* UAP56 has ATP-dependent dsRNA unwinding activity, but apparently it is unable to unwind dsDNA.

### UAP56 interacts directly with mRNA export factors

**Figure 7:**
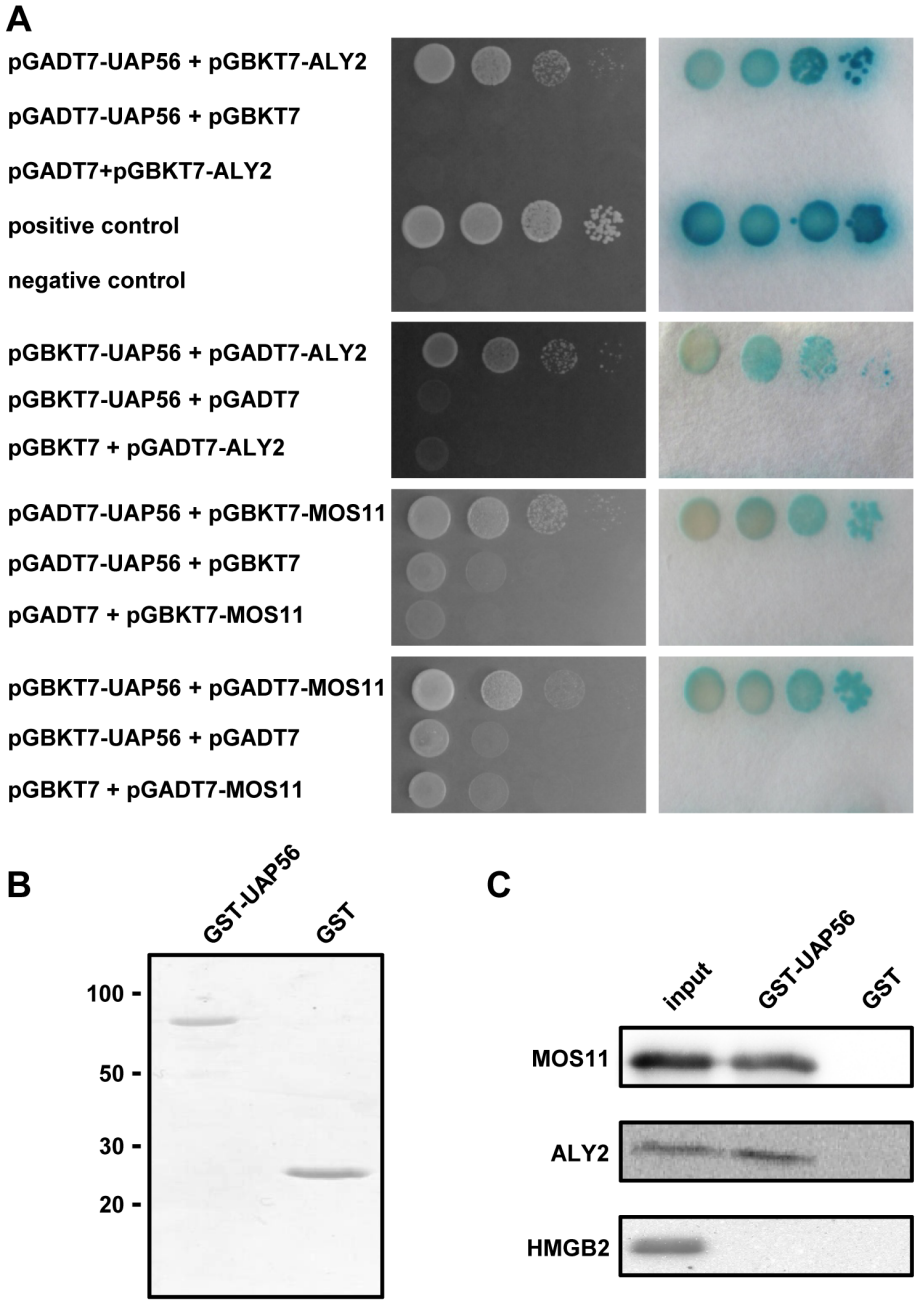
*Arabidopsis* UAP56 interacts with mRNA export factors. **A** Yeast two-hybrid assays. Yeast cells cotransformed with the indicated plasmids were grown on SD/-Leu/-Trp/-His medium (left panels). Cells grown on the same medium were used to detect β-galactosidase reporter gene activity by colony-lift filter assays and incubation of the filters with the X-gal substrate (right panels). **B** Purified GST and GST-UAP56 used for pull-down assays were analysed by SDS-PAGE and Coomassie staining. **C** Pull-down assays with GST and GST-UAP56. Recombinant MOS11, HMGB2 or *in vitro* translated ^35^S-Met-labelled ALY2 were incubated in the presence of ATP with GST and GST-UAP56, which were bound to glutathione cellulose beads. After washing the beads, eluted proteins were analysed by SDS-PAGE. 6xHis-MOS11 and 6xHis-HMGB2 were detected by immunoblotting using an antibody directed against the 6xHis-tag and ALY2 by phosphorimaging. Aliquots of the protein input of MOS11, HMGB2 (10%) and ALY2 (25%) are also shown.

Using the yeast two-hybrid method, we examined whether UAP56 can interact with proteins involved in mRNA export. In *Arabidopsis*, four nuclear ALY proteins were identified [50] and the amino acid sequence of ALY2 is closely related to that of the human mRNA export adaptor ALY. Moreover, the nuclear protein MOS11 was identified as the *Arabidopsis* ortholog of human mRNA export factor CIP29 [41]. Therefore, the coding sequences of UAP56, ALY2 and MOS11 were inserted into the pGBKT7 and pGADT7 vectors that allow expression of the proteins fused to the DNA-binding domain (BD) or the activation domain (AD) of GAL4, respectively. Yeast cells were cotransformed with versions of both vectors and the growth of the yeast strains was scored as well as the expression of the β-galactosidase reporter gene. Each protein was tested both as an AD and BD fusion along with established positive and negative control combinations. In this yeast two-hybrid assay, UAP56 clearly interacted with ALY2 and MOS11 (Figure 7A), as evident from the growth of the cells on selective medium (left panels) and reporter gene expression (right panels), while the negative controls showed only poor growth and no β-galactosidase staining. To validate the interactions seen with the yeast two hybrid method, pull-down experiments were performed with GST and with GST fused to UAP56 (GST-UAP56; Figure 7B). 6xHis-tagged MOS11 or HMGB2 and *in vitro* translated ALY2 were incubated with GST-UAP56 and as a negative control with GST. GST and GST-UAP56 were immobilised on glutathione cellulose beads. After washing the beads, bound proteins were eluted with SDS-loading buffer and analysed by SDS-PAGE. Both MOS11 and ALY2 clearly bound to GST-UAP56, but not to GST, whereas the control protein HMGB2 did not bind to the affinity matrices (Figure 7C). Therefore, in yeast cells and *in vitro* the mRNA export factors MOS11 and ALY2 interacted with *Arabidopsis* UAP56.

## Discussion

In different organisms the RNA helicase UAP56 is a critical factor for pre-mRNA splicing and mRNA export from the cell nucleus. We have identified two *Arabidopsis* genes that code for an identical UAP56 protein, which shares a high degree of amino acid sequence conservation with candidate UAP56 proteins of other plants. Both *UAP56* genes are expressed in *Arabidopsis* seedlings. Upon inactivation of one of the genes in T-DNA insertion mutants, the other gene can compensate for that, resulting in approximately wild type UAP56 levels. In line with that, the mutant plants have wild type appearance. The maintenance of UAP56 wild type levels in the mutant plants indicates the importance of sufficient cellular amount of UAP56. The subcellular localisation of UAP56 fused to GFP was examined in tobacco protoplasts and the native protein was examined in *Arabidopsis* wild type cells using immunofluorescence. As in mammalian cells, *D. melanogaster* and *C. elegans* [8], [11], [47], both experiments demonstrated that *Arabidopsis* UAP56 is a predominantly nuclear protein. Recently, it was found that human UAP56 can shuttle between nucleus and cytosol [51], but from our results there is no clear indication for cytosolic localisation of UAP56 in *Arabidopsis* cells. Rice UAP56 fused to YFP was detected in both nucleus and cytosol of transiently transformed *Arabidopsis* protoplasts [29], which could be due to overexpression of the fusion protein or due to the use of a heterologous expression system. Consistent with a role of UAP56 in mRNA splicing and export, UAP56 was mainly detected in the euchromatic regions of *Arabidopsis* nuclei by immunofluorescence, showing a distribution similar to that of RNAPII. In line with experiments in plant cells and protoplasts [29], [52], but different from the distribution of UAP56 in mammalian and *Drosophila* nuclei [8], [47], *Arabidopsis* UAP56 did not show a speckled distribution in the nucleoplasm. In our experiments also weak nucleolar signals were observed, indicating that low concentrations of UAP56 may occur in the nucleolus. The DEAD-box helicase p68 (which is only distantly related to UAP56) was found to interact with the nucleolar protein fibrillarin [53]. Also consistent with a nucleolar localisation are proteomic studies and the fluorescence microscopic analysis of an UAP56-GFP fusion protein that detected UAP56 in addition to the nucleoplasm in *Arabidosis* nucleoli [52]. Since these authors in addition to UAP56 identified in the nucleolus other proteins known to interact with mRNAs, they suggested that in plants nucleoli may have also functions in mRNA export or surveillance.

The interaction of UAP56 with nucleic acids was studied using EMSA and MST analyses. Both approaches revealed that UAP56 can interact with ssRNA and dsRNA, but also with dsDNA, while ssDNA was not recognised. The interaction with dsDNA was unexpected, since DEAD-box helicases typically are specific for RNA (and RNA-DNA duplexes) [2], [3] and accordingly human UAP56 was shown to interact with various RNA substrates [7]. Previously, two DEAD-box proteins, human DDX3 and yeast DBP9p, which are only distantly related to *Arabidopsis* UAP56 (19.7% and 17.6% amino acid sequence identity, respectively) were reported to interact with both RNA and DNA [54], [55]. Structural studies on DEAD-box helicases in complex with RNA have indicated mechanisms that may explain the specificity for RNA. Thus, the *Drosophila* Vasa helicase forms several specific interactions with 2′-OH groups of ssRNA [56], and in case of the yeast helicase Mss116p, in addition to (non-conserved) contacts with 2′-OH groups of the RNA, the phosphate backbone geometry serves as an important determinant of RNA duplex recognition [57]. Currently, for UAP56 no structure in complex with RNA has been solved explaining the interaction with both RNA and dsDNA.

In line with the immunofluorescence experiments, revealing that UAP56 primarily localises to the euchromatin of *Arabidopsis* nuclei, in ChIP experiments the protein was found to associate with loci transcribed by RNAPII, but not with non-transcribed regions. UAP56 was detected at both intronless genes as well as intron-containing genes. This is in agreement with studies in other organisms, where UAP56 can be recruited in a splicing-dependent manner, but also independent from mRNA splicing [4], [5], [58], [59]. These findings are consistent with the role of UAP56 in splicing and mRNA export [46]. Formaldehyde (used as the standard fixative in ChIP) produces efficiently protein-DNA, but also protein-RNA and protein-protein crosslinks [60]. Therefore, from ChIP experiments it is difficult to judge, whether UAP56 directly interacts with chromatin. In addition to a direct interaction mediated by its DNA binding activity, it is possible that UAP56 is tethered to chromatin by RNA or a partner protein.

Using recombinant UAP56 we have examined the catalytic properties of the *Arabidopsis* enzyme. It displayed basal ATPase activity that is stimulated by the addition of RNA, and ssRNA was more efficient than dsRNA in promoting ATP hydrolysis, which was also observed with human UAP56 [7]. The RNA helicase activity of *Arabidopsis* UAP56 was analysed in unwinding assays, revealing that the unwinding of RNA duplexes is strictly ATP-dependent. Moreover, shorter RNA duplexes were unwound more efficiently than longer (more stable) RNA duplexes and RNA substrates with 5′or 3′overhangs were somewhat less efficiently unwound than a comparable blunt-ended substrate. With human UAP56 blunt-ended substrates and those with (longer) overhangs were unwound with similar efficiency, while the RNA length-dependence of the reaction was also observed [7]. For comparable unwinding efficiency, for instance with the 13-nt substrate, with *Arabidopsis* UAP56 approximately threefold higher protein concentrations were required than with the human enzyme. Most likely this difference is due to the fact that our helicase assays were performed at 28°C, while the assays with human UAP56 were performed at 37°C. Both increased enzymatic activity and lower RNA duplex stability at higher temperature may contribute the different efficiencies. Despite the fact that DEAD-box proteins are bona fide helicases, they unwind RNA rather inefficiently when helix length rises above 10–15 bp, but helical elements within structured RNA substrates are rarely longer than 10 bp [2], [3]. Our EMSA and MST analyses revealed that *Arabidopsis* UAP56 in addition to RNA can bind dsDNA, but not ssDNA. Therefore, we tested whether dsDNA stimulates the ATPase activity of UAP56, and we observed a similar efficiency as seen with ssRNA. However, in our helicase assay UAP56 was unable to unwind dsDNA. The two DEAD-box proteins, human DDX3 and yeast DBP9p (73 and 68 kDa, respectively), which also interact with RNA and DNA, were found to unwind both dsRNA and dsDNA [54], [55], but these proteins are significantly bigger than UAP56, having additional domain(s).

Both yeast two-hybrid and GST pull-down experiments demonstrated that *Arabidopsis* UAP56 can interact with the mRNA export adaptors ALY2 and MOS11, suggesting that also in plants the RNA helicase in combination with the corresponding export factors is involved in mRNA export from the nucleus. We have tested ALY2, because according to sequence alignments it is a close relative of human ALY [50], while MOS11 most likely is the *Arabidopsis* ortholog of human CIP29, and mutant *mos11* cells display mRNA export defects [41]. The export adaptors ALY (yYRA1) and CIP29 were found to be recruited by human UAP56 (ySUB2) to mRNAs [12], [13], finally resulting in a “hand-over” of the mRNA to export receptors mediating translocation of the mRNPs through the nuclear pore complexes [15], [16]. UAP56 is thought to be involved in various steps of remodelling of the mRNPs during maturation and export [15], [16], [46]. In mammals, there is a second DEAD-box RNA helicase URH49, sharing ∼90% amino acid sequence identity with UAP56. Apparently, both UAP56 and URH49 can interact with CIP29, whereas ALY seems to interact with UAP56 and not with URH49 [13], [61]. In *Arabidopsis*, according to database searches, there is no second helicase closely related to UAP56. Therefore, it is likely that *Arabidopsis* UAP56 is a functional interactor of both ALY2 and MOS11.

Consistent with its essential role in splicing and mRNA export, depletion as well as overexpression of UAP56 in mammals, *Drosophila, Caenorhabditis* and yeast severely affected mRNA export and cell growth/survival [8], [11], [12], [62], [63]. In yeast, for instance, depending on the strain background inactivation of the *SUB2* gene can be lethal, while overexpression of SUB2 impairs mRNA export in a dominant negative manner presumably by titrating mRNA export factors [12], [63]. In *Arabidopsis* however, the overexpression of UAP56 does not result in altered growth and development of the transgenic plants. In rice, down-regulation of the *UAP56* (*AIP1/2*) transcript levels by amiRNA resulted in reduced fertility, presumably due to defects in anther/pollen development [29]. The authors suggest that AIP1 negatively controls the expression of the *CP1* gene (encoding a Cys protease) by specifically binding to the *CP1* promoter, thereby regulating tapetum degeneration in anthers. Due to the close proximity of the two *Arabidopsis UAP56* genes on chromosome 5, it is unlikely to obtain double mutants (by crossing of the single mutants analysed here) that do not express UAP56. We have attempted generating *Arabidopsis* plants with down-regulated expression of *UAP56* using the RNAi strategy. So far we were unable obtaining plants with significantly reduced expression of *UAP56* (data not shown), but this is clearly a goal of future experiments.

## Supporting Information

Figure S1 **Multiple sequence alignment of (putative) UAP56 sequences from different organisms.**
(PDF)

Figure S2 **Sequence similarity of UAP56 sequences.**
(PDF)

Figure S3 **Characterisation of** ***Arabidopsis*** **T-DNA insertion mutants.**
(PDF)

Figure S4 **Phenotypic analysis of T-DNA insertion lines** ***uap56a-3*** **and** ***uap56b-1*** **.**
(PDF)

Figure S5 **Analysis of plants overexpressing** ***UAP56*** **.**
(PDF)

Table S1 **Oligonucleotide primers used in this study and construction of plasmids.**
(PDF)

Table S2 **RNA and DNA oligonucleotides used in different biochemical assays of this study.**
(PDF)
