# Supplementary material for: Arabidopsis DEAD-Box RNA Helicase UAP56 Interacts with Both RNA and DNA as well as with mRNA Export Factors
Source: PLoS One. 2013 Mar 26;8(3):e60644. doi: 10.1371/journal.pone.0060644 (PMC3608606; doi:10.1371/journal.pone.0060644)
Supplement: Figure S1 — Multiple sequence alignment of (putative) UAP56 sequences from different organisms. (PDF) [file pone.0060644.s001.pdf]

```
AtUAP56         --MGDARDNEAYEEELLDYEEEDEKV------------------PDS---GNKVNGEAVK
OsAIP2          MGEAEVKDNEVYEEDLVDYEEEVENG------------------ADGGAAAANASADVVK
OsAIP1          MAEAEVKDNEVYEEDLVDYEEEVENG------------------TDGGANAANASADVVK
SmUAP56         --MGEAKENDTYQEELLDYEEEEEAA------------------PDAV--AAKGAAETVK
PpUAP56         --MG---EPYPETEELLDYEEEEEAA------------------PDAV--AAKTNGETVK
CrUAP56         ------MADPANEEELLDYEEDDVAE------------------AEAV-----GKAGQEK
HsUAP56         ------MAENDVDNELLDYEDDEVET------------------AAGGDGAEAPAKKDVK
DmHEL           ---------MADNDDLLDYEDEEQTE------------------TTAVENQ-EAPKKDVK
CeUAP56         ----------MEEEQLLDYEEEQEEI------------------QDKQPEVGGGDARKTK
ScSUB2          -------MSHEGEEDLLEYSDNEQEIQIDASKAAEAGETGAATSATEGDNNNNTAAGDKK
                             ::*::*.::                                     *

AtUAP56         KGYVGIHSSGFRDFLLKPELLRAIVDSGFEHPSEVQHECIPQAILGMDVICQAKSGMGKT
OsAIP2          KGYVGIHSSGFRDFLLKPELLRAIQDCGFEHPSEVQHECIPQAILGMDVICQAKSGMGKT
OsAIP1          KGYVGIHSSGFRDFLLKPELLRAIQDCGFEHPSEVQHECIPQAILGMDVICQAKSGMGKT
SmUAP56         KGYVGIHSSGFRDFLLKPELVRAIVDCGFEHPSEVQFECIPQAILGMDVICQAKSGMGKT
PpUAP56         KGYVGIHSSGFRDFLLKPELLRAIVDCGFEHPSEVQHECIPQAILGMDVICQAKSGMGKT
CrUAP56         KGYVGIHATGFKELMLKPELLQAIADCGFEHPSEVQHECIPHAILGNDVLCQAKSGMGKT
HsUAP56         GSYVSIHSSGFRDFLLKPELLRAIVDCGFEHPSEVQHECIPQAILGMDVLCQAKSGMGKT
DmHEL           GTYVSIHSSGFRDFLLKPEILRAIVDCGFEHPSEVQHECIPQAVLGMDILCQAKSGMGKT
CeUAP56         GTYASIHSSGFRDFLLKPEILRAIGDCGFEHPSEVQHECIPQAILGMDVVCQAKSGMGKT
ScSUB2          GSYVGIHSTGFKDFLLKPELSRAIIDCGFEHPSEVQQHTIPQSIHGTDVLCQAKSGLGKT
                  *..**::**::::****: :** *.********* . **::: * *::******:***
                                              Q                         I

AtUAP56         AVFVLSTLQQIEPSPG-QVSALVLCHTRELAYQICNEFVRFSTYLPDTKVSVFYGGVNIK
OsAIP2          AVFVLSSLQQIDPVAG-QVGALVLCHTRELAYQICHEFERFSKYLPEVKVAVFYGGVHIK
OsAIP1          AVFVLSSLQQIDPVAG-QVGALVLCHTRELAYQICHEFERFSKYLPEVKVAVFYGGVHIK
SmUAP56         AVFVLSTLQQIEPLKG-QVAALVLCHTRELAYQICHEFERFSTYLPDIKVAVFYGGVNIK
PpUAP56         AVFVLSTLQQIEPVAG-QVAALVLCHTRELAYQICHEFERFSTYLPDIKVAVFYGGVNIK
CrUAP56         AVFVLSILQQLDPKAN-ECHAIILCHTRELAFQICHEFTRFSARMKGVTIGNFYGGIPVT
HsUAP56         AVFVLATLQQLEPVTG-QVSVLVMCHTRELAFQISKEYERFSKYMPNVKVAVFFGGLSIK
DmHEL           AVFVLATLQQLEPSDNNTCHVLVMCHTRELAFQISKEYERFSKYMPTVKVAVFFGGMAIQ
CeUAP56         AVFVITTLQQLEPVDG-EVSVVCMCHTRELAFQISKEYERFSKYLPGVKVAVFFGGMAIK
ScSUB2          AVFVLSTLQQLDPVPG-EVAVVVICNARELAYQIRNEYLRFSKYMPDVKTAVFYGGTPIS
                ****:: ***::*       .: :*.:****:** .*: ***  :   . . *:**  :
                                           Ia                          GG

AtUAP56         IHKDLLKN--ECPHIVVGTPGRVLALAREKDLSLKNVRHFILDECDKMLESLDMRRDVQE
OsAIP2          KHKDLLKN--DCPHIVVGTPGRILALAREKDLSLKNVRHFILDECDKMLDSLDMRRDVQE
OsAIP1          KHKDLLKN--DCPHIVVGTPGRILALAREKDLSLKNVRHFILDECDKMLDSLDMRRDVQE
SmUAP56         THKDLLKN--ECPHIVVGTPGRILQLTREKELSLKGVRHFILDECDKMLESLDMRRDVQE
PpUAP56         THKDLLKN--ECPHIVVGTPGRILGLARDKDLSLKSVRHFILDECDKMLESLDMRRDVQE
CrUAP56         QNKETLKK--AVPNIVVGTPGRIKQLAKEGALPLKHVRFFVLDECDKMLEKLDMRADCQE
HsUAP56         KDEEVLKK--NCPHIVVGTPGRILALARNKSLNLKHIKHFILDECDKMLEQLDMRRDVQE
DmHEL           KDEETLKS--GTPHIVVGTPGRILALIRNKKLNLKLLKHFVLDECDKMLEQLDMRRDVQE
CeUAP56         KDEERLAN--DCPHIVVGTPGRMLALARSGKLKLDKVKYFVLDECDKMIGDADMRRDVQE
ScSUB2          KDAELLKNKDTAPHIVVATPGRLKALVREKYIDLSHVKNFVIDECDKVLEELDMRRDVQE
                 . : * .    *.***.****:  * :.  : *. :: *::*****:: . *** * **
                                    Ib                        DECD (II)

AtUAP56         IFKMTPHDKQVMMFSATLSKEIRPVCKKFMQDPMEIYVDDEAKLTLHGLVQHYIKLSEME
OsAIP2          IFKMTPHDKQVMMFSATLSKEIRPVCKKFMQDPMEIYVDDEAKLTLHGLVQHYIKLSEAE
OsAIP1          IFKMTPHDKQVMMFSATLSKEIRPVCKKFMQDPMEIYVDDEAKLTLHGLVQHYIKLSEAE
SmUAP56         IFKLTPHDKQVMMFSATLSKEIRPVCKRFMQDPMEIYVDDEAKLTLHGLVQHYIKLSEAE
PpUAP56         IFKLTPHDKQVMMFSATLSKEIRPVCKRFMQDPMEIYVDDEAKLTLHGLVQHYIKLSEVE
CrUAP56         IFKLTPHEKQVMMFSATLNQDMRGVCKKFMTNPQEVYVDDESKLTLHGLVQHYVMLHEEE
HsUAP56         IFRMTPHEKQVMMFSATLSKEIRPVCRKFMQDPMEIFVDDETKLTLHGLQQYYVKLKDNE
DmHEL           IFRSTPHGKQVMMFSATLSKDIRPVCKKFMQDPMEVYVDDEAKLTLHGLQQHYVNLKENE
CeUAP56         IVKMTPQQKQVMMFSATLPKELRTVCKRFMQDPMEVYVDDEAKLTLHGLQQHYVKLKEAE
ScSUB2          IFRATPRDKQVMMFSATLSQEIRPICRRFLQNPLEIFVDDEAKLTLHGLQQYYIKLEERE
                *.: **: ********** :::* :*::*: :* *::****:******* *:*: * : *
                               III
```

```
AtUAP56      KNRKLNDLLDALDFNQVVIFVKSVSRAAELNKLLVECNFPSICIHSGMSQEERLTRYKSF
OsAIP2       KNRKLNDLLDALDFNQVVIFVKSVSRAAELNKLLCECNFPAISIHSGMTQEERLTRYKNF
OsAIP1       KNRKLNDLLDALDFNQVVIFVKSVSRAAELNKLLCECNFPAISIHSGMTQEERLTRYKNF
SmUAP56      KNRKLNDLLDALDFNQVVIFVKSVNRAAELNKLLVECNFPSICIHSGMSQEERLTRYKGF
PpUAP56      KNRKLNDLLDALDFNQVVIFVKSVSRAAELNKLLVECNFPSICIHSGMSQEERLTRYKGF
CrUAP56      KNRKLNDLLDALDFNQVVIFVKSVARAKMLNTLLNECNFPSVCIYGGMDQEERIKVYKHF
HsUAP56      KNRKLFDLLDVLEFNQVVIFVKSVQRCIALAQLLVEQNFPAIAIHRGMPQEERLSRYQQF
DmHEL        KNKKLFELLDVLEFNQVVIFVKSVQRCVALSQLLTEQNFPAIGIHRGMTQEERLNRYQQF
CeUAP56      KNRRLLNLLDALEFNQVVIFVKAVKRCEALHQLLTEQNFPSIAIHRQMAQEERLSRYQAF
ScSUB2       KNRKLAQLLDDLEFNQVIIFVKSTTRANELTKLLNASNFPAITVHGHMKQEERIARYKAF
             **::* :*** *:****:****:. *.  *  **   ***:: ::  * ****:  *: *

                       IV                                 QxxR
```

```
AtUAP56      KEGHKRILVATDLVGRGIDIERVNIVINYDMPDS-------------ADTYLHRVGRAGR
OsAIP2       KEGHKRILVATDLVGRGIDIERVNIVINYDMPDS-------------ADSYLHRVGRAGR
OsAIP1       KEGHKRILVATDLVGRGIDIERVNIVINYDMPDS-------------ADSYLHRVGRAGR
SmUAP56      KEGHKRILVATDLVGRGIDIERVNIVINYDMPES-------------ADTYLHRVGRAGR
PpUAP56      KEGHKRILVATDLVGRGIDIERVNIVINYDMPDS-------------ADTYLHRVGRAGR
CrUAP56      KEGKHRILVATDLVGRGIDIERVNIVINYDMPESDDKSKGESKHGNGADTYLHRVGRAGR
HsUAP56      KDFQRRILVATNLFGRGMDIERVNIAFNYDMPED-------------SDTYLHRVARAGR
DmHEL        KDFQKRILVATNLFGRGMDIERVNIVFNYDMPED-------------SDTYLHRVARAGR
CeUAP56      KDFQKRILVATDLFGRGMDIERVNIVFNYDMPED-------------SDSYLHRVARAGR
ScSUB2       KDFEKRICVSTDVFGRGIDIERINLAINYDLTNE-------------ADQYLHRVGRAGR
             *: .:** *:*::.***:****:*:.:***: :.             :* *****.****

                    V                                          VI
```

```
AtUAP56      FGTKGLAITFVASASDSEVLNQVQERFEVDIKELPEQ-IDTSTYMPS-------
OsAIP2       FGTKGLAITFVSSASDSDVLNQVQERFEVDIKELPEQ-IDTSTYMPS-------
OsAIP1       FGTKGLAITFVSSASDSDVLNQVQERFEVDIKELPEQ-IDTSTYMPS-------
SmUAP56      FGTKGLAITFVASSTDSNVLNQVQERFEVDIKELPEQ-IDTSTYMPS-------
PpUAP56      FGTKGLAITFVASAPDSNVLNQVQERFEVDIKELPEQ-IDTTTYSKIINLNICI
CrUAP56      FGTKGLAITFVSSQEDSAVLNAVQDRFDVDIKPLPEK-IDASTYMNTN------
HsUAP56      FGTKGLAITFVSDENDAKILNDVQDRFEVNISELPDE-IDISSYIEQTR-----
DmHEL        FGTKGLAITFVSDENDAKILNEVQDRFDVNISELPEE-IDLSTYIEGR------
CeUAP56      FGTKGLAITFVSDENDAKTLNSVQDRFDISITELPEK-IDVSTYIEGRTN----
ScSUB2       FGTKGLAISFVSSKEDEEVLAKIQERFDVKIAEFPEEGIDPSTYLNN-------
             ********:**:.  *   *  :*:**::.*  :*:: ** ::*
```

Figure. S1. Multiple sequence alignment of (putative) UAP56 sequences from different organisms. The amino acid sequences of different species (*At: Arabidopsis thaliana, Os: Oryza sativa, Pp: Physcomitrella patens, Sm: Selaginella moellendorffii, Cr: Chlamydomonas reinhardtii, Hs: Homo sapiens, Dm: Drosophila mleanogaster, Ce: Caenorhabditis elegans, Sc: Saccharomyces cerevisiae)* were aligned using Clustal Omega (http://www.ebi.ac.uk/Tools/msa/clustalo/). Characteristic helicase motifs (Jarmoskaite and Russell, 2011; Linder and Jankowsky, 2011; Shen, 2009) are indicated by black bars below the sequences.
