# Supplementary material for: Arabidopsis DEAD-Box RNA Helicase UAP56 Interacts with Both RNA and DNA as well as with mRNA Export Factors
Source: PLoS One. 2013 Mar 26;8(3):e60644. doi: 10.1371/journal.pone.0060644 (PMC3608606; doi:10.1371/journal.pone.0060644)
Supplement: Figure S2 — Sequence similarity of UAP56 sequences. (PDF) [file pone.0060644.s002.pdf]

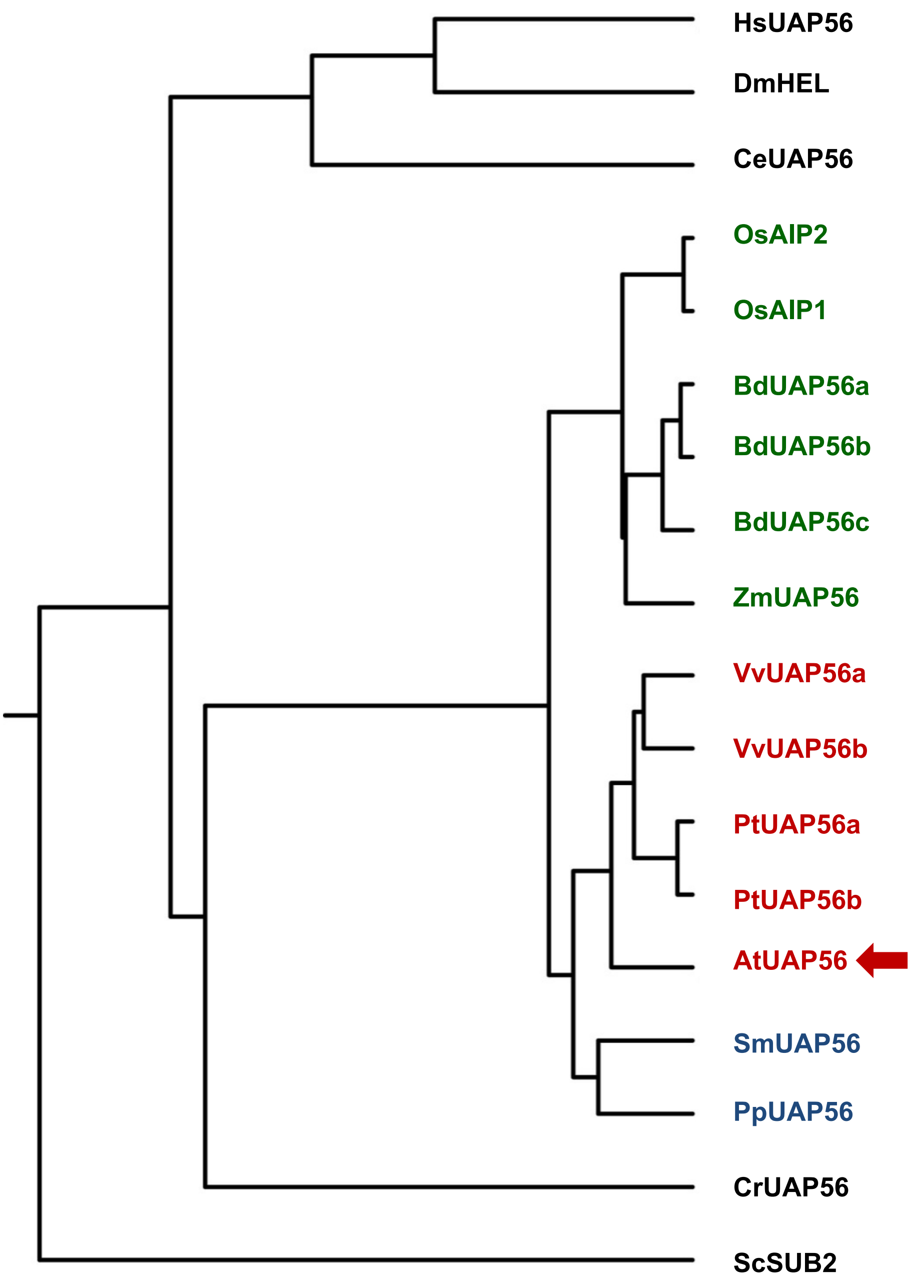

Figure S2. Sequence similarity of UAP56 sequences. The amino acid sequences of (putative) UAP56 proteins from various organisms (At, *Arabidopsis thaliana*; Bd, *Brachypodium distachyon*; Ce, *Caenorhabditis elegans*; Cr, *Chlamydomonas reinhardtii; Dm, Drosophila melanogaster; Hs, Homo sapiens; Os, Oryza sativa; Pp, Physcomitrella patens; Pt, Populus trichocarpa; Sc, Saccharomyces cerevisiae; Sm, Selaginella moellendorfii; Vv, Vitis vinifera; Zm, Zea mays)* were aligned by multiple sequence alignment (Clustal W) that served to cluster the sequences (unweighted pair group method with arithmetic mean) in a rooted tree (http://www.genome.jp/tools/clustalw/). The sequences of monocots are indicated in green and those of dicot plants in red, and the *Arabidopsis* UAP56 studied here is indicated by an arrow. The close similarity of the monocot and dicot sequences with those of *Selaginella* and *Physcomitrella* (in blue) is evident, as well as the more distant relation of the algal, yeast and animal/human sequences (in black).
