# Supplementary material for: Arabidopsis DEAD-Box RNA Helicase UAP56 Interacts with Both RNA and DNA as well as with mRNA Export Factors
Source: PLoS One. 2013 Mar 26;8(3):e60644. doi: 10.1371/journal.pone.0060644 (PMC3608606; doi:10.1371/journal.pone.0060644)
Supplement: Figure S3 — Characterisation of Arabidopsis T-DNA insertion mutants. (PDF) [file pone.0060644.s003.pdf]

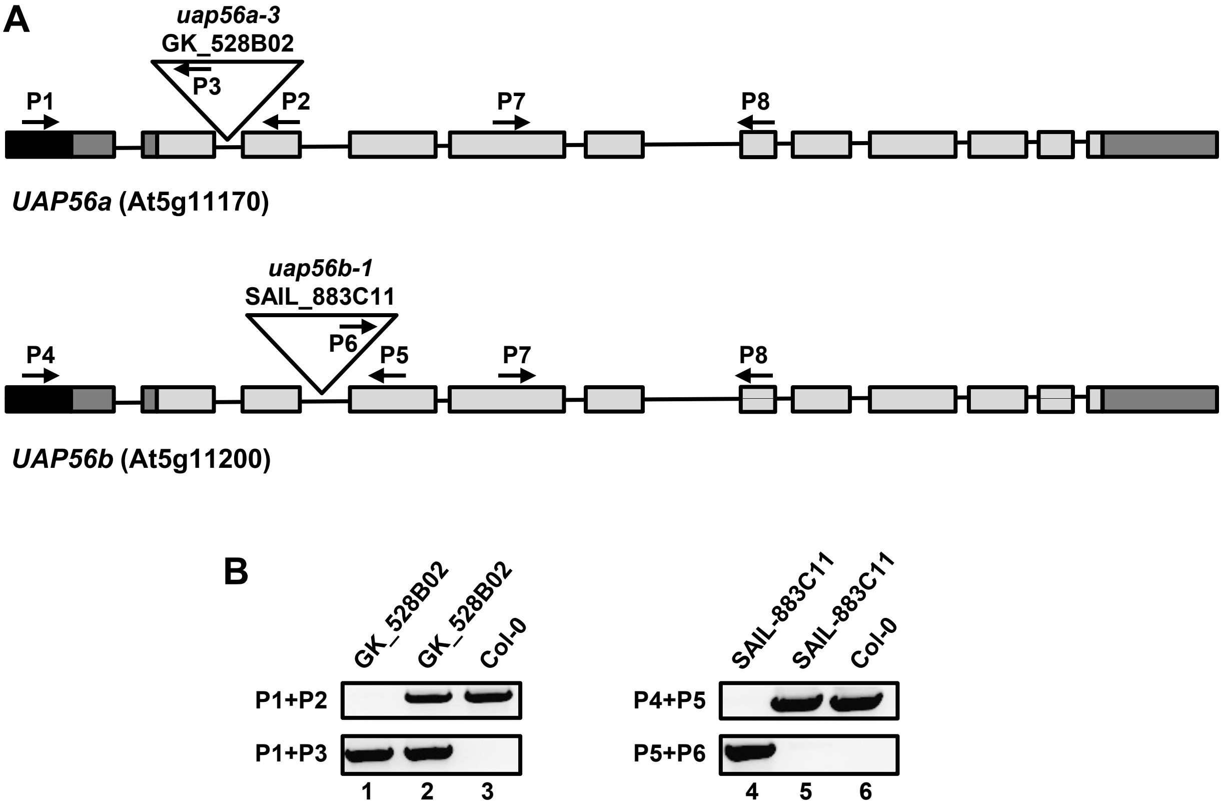

Figure S3. Characterisation of *Arabidopsis* T-DNA insertion mutants. (A) Schematic representation of the two *UAP56* genes. Gray boxes represent exons (UTRs dark gray fill, CDS light gray fill), while black boxes indicate upstream sequences and lines indicate introns (not drawn to scale). The triangles indicate the T-DNA insertions in the two genes and oligonucleotide primers used for molecular characterisation of the loci are indicated by arrows (cf. Table S1). (B) PCR analysis of genomic DNA isolated from a segregating population of seedlings documenting that we were able to isolate plants homozygous for the T-DNA insertions *uap56a-3* (lane 1) and *uap56b-1* (lane 4). Examples of segregating plants hemizygous (lane 2) or lacking (lane 5) the insertions are shown as well as Col-0 controls (lanes 3 and 6).
