# Supplementary material for: Arabidopsis DEAD-Box RNA Helicase UAP56 Interacts with Both RNA and DNA as well as with mRNA Export Factors
Source: PLoS One. 2013 Mar 26;8(3):e60644. doi: 10.1371/journal.pone.0060644 (PMC3608606; doi:10.1371/journal.pone.0060644)
Supplement: Figure S4 — Phenotypic analysis of T-DNA insertion lines uap56a-3 and uap56b-1 . (PDF) [file pone.0060644.s004.pdf]

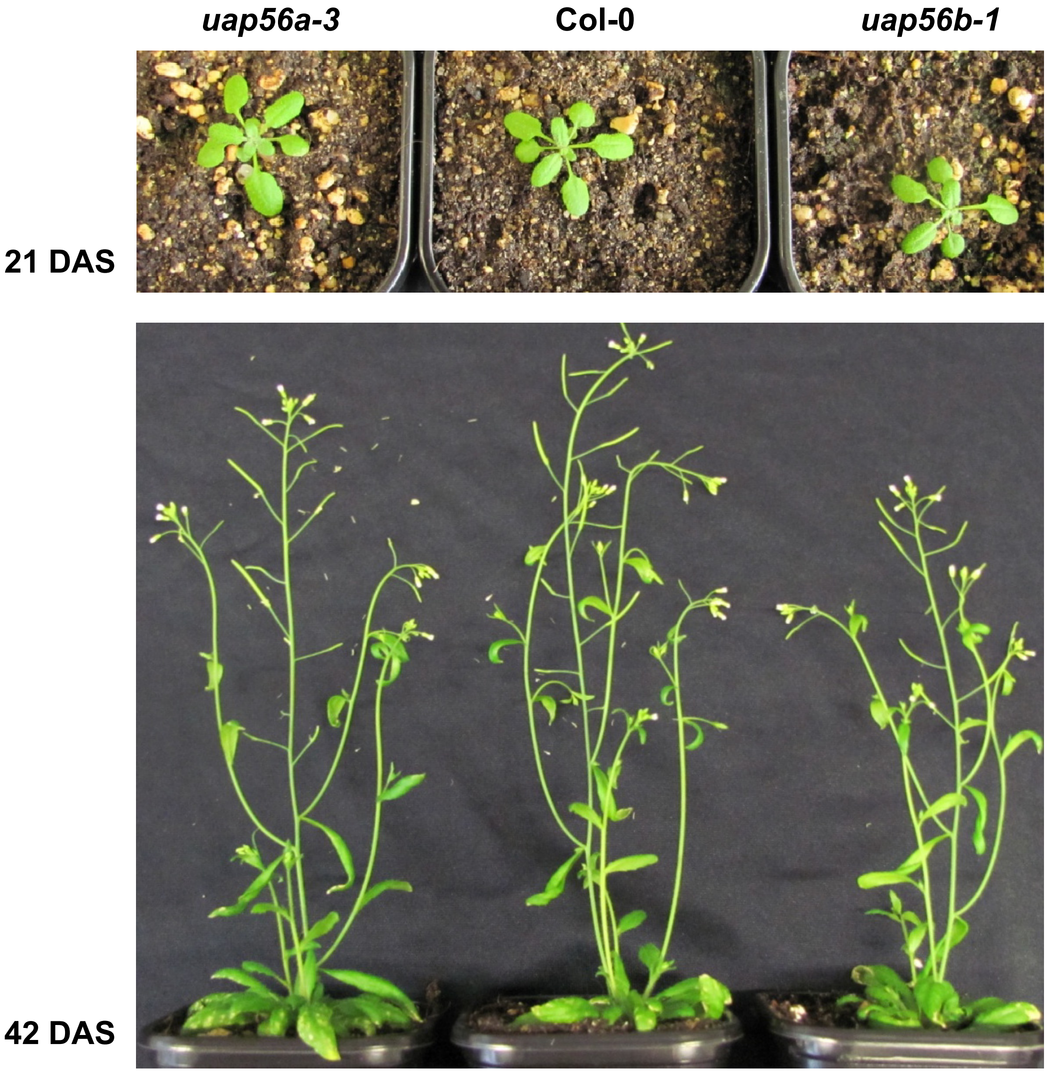


**Figure S4. Phenotypic analysis of T-DNA insertion lines *uap56a-3* and *uap56b-1*.** Mutant plants along with the wild type control Col-0 were grown for different time (21, 42 DAS) under long-day conditions and images of representative indviduals are shown.
