# Supplementary material for: Arabidopsis DEAD-Box RNA Helicase UAP56 Interacts with Both RNA and DNA as well as with mRNA Export Factors
Source: PLoS One. 2013 Mar 26;8(3):e60644. doi: 10.1371/journal.pone.0060644 (PMC3608606; doi:10.1371/journal.pone.0060644)
Supplement: Figure S5 — Analysis of plants overexpressing UAP56 . (PDF) [file pone.0060644.s005.pdf]

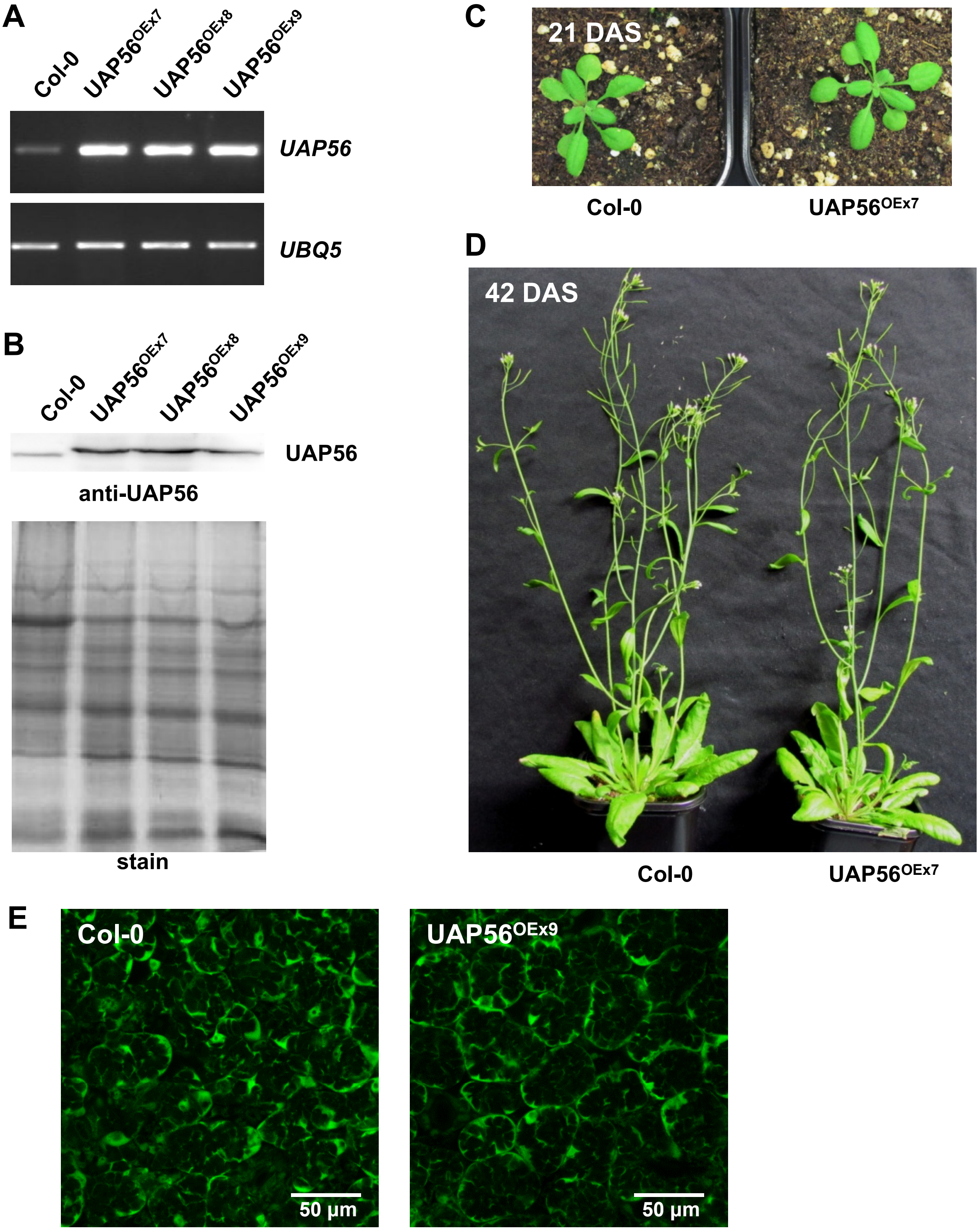

Figure S5. Analysis of plants overexpressing *UAP56*. (**A**) Transcript level of three independent overexpression lines (expressing UAP56 under control of the CaMV 35S promoter) relative to the non-transformed control line (Col-0). Amplification of the *UAP56* transcript is shown along with the reference *UBQ5*. (**B**) Protein level of the overexpression lines examined by immunoblot analysis of protein extracts using the UAP56 antibody (top panel), detecting the wild type protein in Col-0 and myc-tagged UAP56 in the overexpressing plants. A Coomassie stain of the protein extracts is also shown (bottom panel). (**C,D**) Overexpressing plants and Col-0 were grown for different time (21, 42 DAS) under long-day conditions and images of representative indviduals are shown. (**E**) Whole mount *in situ* mRNA localization of 6-day-old seedlings probed with Alexa 488-labeled oligo d(T).
