# Supplementary material for: Arabidopsis DEAD-Box RNA Helicase UAP56 Interacts with Both RNA and DNA as well as with mRNA Export Factors
Source: PLoS One. 2013 Mar 26;8(3):e60644. doi: 10.1371/journal.pone.0060644 (PMC3608606; doi:10.1371/journal.pone.0060644)
Supplement: Table S1 — Oligonucleotide primers used in this study and construction of plasmids. (PDF) [file pone.0060644.s006.pdf]

Table S1. Oligonucleotide primers used in this study and construction of plasmids

No.	primer	use	plasmid	restr. site
P1	GTTCGTATTGACTCGAACC	Genotyping, T-DNA insertion *UAP56*		
P2	AGATGGATGTTCAAATCCAGA	Genotyping, T-DNA insertion *UAP56*		
P3	ATATTGACCATCATACTCATTGC	Genotyping, T-DNA insertion *UAP56*		
P4	CCCCTGCGGTATAAGACG	Genotyping, T-DNA insertion *UAP56*		
P5	CTTTGCTTGGCAGATGACAT	Genotyping, T-DNA insertion *UAP56*		
P6	GCCTTTTCAGAAATGGATAAATAGCCTTGCTTCC	Genotyping, T-DNA insertion *UAP56*		
P7	TGTGATAAAATGCTCGAGTCA	RT-PCR, *UAP56*,fw		
P8	CTGGACAAGCCCATGAAGAG	RT-PCR, *UAP56*,rv		
	CTTGAAGACGGCCGTACCCTC	RT-PCR, *UBQ5*,fw		
	CGCTGAACCTTTCAAGATCCATCG	RT-PCR, *UBQ5*,rv		
	TCCCCCGGGCATGGGGAGACGCTAGAGAC	Insertion of *UAP56*-CDS in pGreenII0229myc,fw	pGreenII0229myc-UAP56	SmaI
	GGAATTCTTAAGAAGGCATGTAGGTTG	Insertion of *UAP56*-CDS in pGreenII0229myc,rv		EcoRI
	CGCAAGACCCTTCCTCTATA	Genotyping, overexpressing plants,fw		
	CTTTGCTTGGCAGATGACAT	Genotyping, overexpressing plants,rv		
	GGAATTCCATATGATGGGAGACGCTAG	Insertion of *UAP56*-CDS pGBKT7,fw	pGBKT7-UAP56	NdeI
	TTCTGCAGTTAAGAAGGCATGTA	Insertion of *UAP56*-CDS pGBKT7,rv		BamHI
	GGAATTCCATATGATGGGAGACGCTAG	Insertion of *UAP56*-CDS in pGADT7,fw	pGADT7-UAP56	NdeI
	GGAATTCTTAAGAAGGCATGTAGGTTG	Insertion of *UAP56*-CDS in pGADT7,rv		EcoRI
	GGAATTCCATATGATGTCAGGTGGCTTAGAT	Insertion of *ALY2* in pGADT7/pGBKT7,fw	pGADT7-ALY2	NdeI
	CGGGATCCCTAACTTGTTTCCATTGC	Insertion of *ALY2* in pGADT7/pGBKT7,rv	pGBKT7-ALY2	BamHI
	GGAATTCCATATGATGGCGACCAACGGA	Insertion of *MOS11* in pGBKT7/pGADT7,fw	pGADT7-MOS11	NdeI
	CGGGATCCTTAGGCAGCGCTTCC	Insertion of *MOS11* in pGBKT7/pGADT7,rv	pGBKT7-MOS11	BamHI
	AACTGCAGATGGGAGACGCTAGA	Insertion of *UAP56*-CDS in pQE9,fw	pQE9-UAP56	PstI
	CCCAAGCTTTTAAGAAGGCATGTA	Insertion of *UAP56*-CDS in pQE9,rv		HindIII
	CGGGATCCATGTCAGGTGGCTTA	Insertion of *ALY2*-CDS in pBluescript II SK (+)	pBluescript-ALY2	BamHI
	ACGCGAGCTCCTAACTTGTTTCCAT	Insertion of *ALY2*-CDS in pBluescript II SK (+)		SacI
	GCTCTAGAATGGGAGACGCTAGA	Insertion of *UAP56*-CDS in pGEX-5X-1	pGEX-5X-1-UAP56	XbaI
	ATAAGAATGCGGCCGCTTAAGAAGGCATGTA	Insertion of *UAP56*-CDS in pGEX-5X-1		NotI
	CGGAATTCATGGGAGACGCTAGA	Insertion of *UAP56*-CDS in 3'GFP vector	p3´GFP-UAP56	XbaI
	TCCCCCGGGTTAAGAAGGCATGTA	Insertion of *UAP56*-CDS in 3'GFP vector		SmaI
	GAAGGCGAAGATCCAAGACAAGGAA	ChIP, At3g62250		
	GGAGGACGAGATGAAGCGTCGA	ChIP, At3g62250		
	GCATGCGTTGTGGTTTTATG	ChIP, At1g49240		
	TCGACAGAAGCGAGAAGAATC	ChIP, At1g49240		
	TTCTTCCACGACTGTTTCCCTAACG	ChIP, At3g28200		
	CGACGGTGATTAGAAGATCACGTGT	ChIP, At3g28200		
	TCAACACGTGTCCTCACGTTTCTG	ChIP, At1g71695		
	AGGATGGCAGCGGCTAAACCA	ChIP, At1g71695		
	TCAGACGACTCAGATTTCATCCGTAC	ChIP, At1g23205		
	TGGTGAGCACCATCTTCGTTGC	ChIP, At1g23205		
	CCATCTCCATCAACATAAGCCCCAA	ChIP, At2g26440		
	CGTTAACACCGTCTTGGATCTTGGA	ChIP, At2g26440		
	GCACTACATACAGTTGTGTCGGAGT	ChIP, At1g16030		
	GTATGTCACTCTGAACAGAAGGATCAGA	ChIP, At1g16030		
	CACTGACAGCGAGCGTCTCATC	ChIP, At3g12580		
	CACACTAAACCGGCTCTGTTTCGA	ChIP, At3g12580		
	TGCAAATCGTTCTCTCCTTG	ChIP, Ta2		
	AAACGATGCGTTGGGATAGGTC	ChIP, Ta2		
	GATTCTTACTGTAAAGAACATGGCATTGAGAGA	ChIP. Ta3		
	TCCAATTTCCTGAGGTGCTTGTAACC	ChIP, Ta3		
