# Supplementary material for: Arabidopsis DEAD-Box RNA Helicase UAP56 Interacts with Both RNA and DNA as well as with mRNA Export Factors
Source: PLoS One. 2013 Mar 26;8(3):e60644. doi: 10.1371/journal.pone.0060644 (PMC3608606; doi:10.1371/journal.pone.0060644)
Supplement: Table S2 — RNA and DNA oligonucleotides used in different biochemical assays of this study. (PDF) [file pone.0060644.s007.pdf]

**Table S2. RNA and DNA oligonucleotides used in different biochemical assays of this study**

| Name[1] | Sequence 5´- 3´ |
|---|---|
| **R13** | GCUUUACGGUGCU |
| **R13C** | AGCACCGUAAAGC |
| **R13ds** | GCUUUACGGUGCU<br>CGAAAUGCCACGA |
| **R16** | ACUAGCACCGUAAAGC |
| **R16C** | GCUUUACGGUGCUAGU |
| **R16ds** | ACUAGCACCGUAAAGC<br>UGAUCGUGGCAUUUCG |
| **R13/R16 (5´overhang)** | GCUUUACGGUGCU<br>CGAAAUGCCACGAUCA |
| **R13C/R16C (3´overhang)** | AGCACCGUAAAGC<br>UGAUCGUGGCAUUUCG |
| **R25** | AAAACAAAAUAGCACCGUAAAGCAC |
| **R25C** | GUGCUUUACGGUGCUAUUUUGUUUU |
| **R25ds** | AAAACAAAAUAGCACCGUAAAGCAC<br>UUUUGUUUUAUCGUGGCAUUUCGUG |
| **R29** | CCUCCUUUUUUCUUUUUUUUUUUUUUUCU |
| **R29C** | AGAAAAAAAAAAAAAAAGAAAAAAGGAGG |
| **R29ds** | CCUCCUUUUUUCUUUUUUUUUUUUUUUCU<br>GGAGGAAAAAAGAAAAAAAAAAAAAAAGA |
| **D13ds** | GCTTTACGGTGCT<br>CGAAATGCCACGA |
| **D25** | AAAACAAAATAGCACCGTAAAGCAC |
| **D25C** | GTGCTTTACGGTGCTATTTTGTTTT |
| **D25ds** | AAAACAAAATAGCACCGTAAAGCAC<br>TTTTGTTTTATCGTGGCATTTCGTG |
| **D29** | CCTCCTTTTTTCTTTTTTTTTTTTTTTTCT |
| **D29C** | AGAAAAAAAAAAAAAAAGAAAAAAGGAGG |
| **D29ds** | CCTCCTTTTTTCTTTTTTTTTTTTTTTCT<br>GGAGGAAAAAAGAAAAAAAAAAAAAAAGA |

[1] R indicates an RNA and D indicates a DNA oligonucleotide.
